# Computational singular perturbation analysis of brain lactate metabolism

**DOI:** 10.1371/journal.pone.0226094

**Published:** 2019-12-17

**Authors:** Dimitris G. Patsatzis, Efstathios-Al. Tingas, Dimitris A. Goussis, S. Mani Sarathy

**Affiliations:** 1 King Abdullah University of Science and Technology (KAUST), Clean Combustion Research Center (CCRC), Thuwal, Saudi Arabia; 2 Department of Mechanics, School of Applied Mathematics and Physical Sciences, National Technical University of Athens (NTUA), Athens, Greece; 3 Perth College, University of the Highlands and Islands, Crieff Rd, Perth PH1 2NX, United Kingdom; 4 Department of Mechanical Engineering, Khalifa University of Science, Technology and Research (KUSTAR), Abu Dhabi, United Arab Emirates; University of Connecticut School of Medicine, UNITED STATES

## Abstract

Lactate in the brain is considered an important fuel and signalling molecule for neuronal activity, especially during neuronal activation. Whether lactate is shuttled from astrocytes to neurons or from neurons to astrocytes leads to the contradictory *Astrocyte to Neuron Lactate Shuttle* (ANLS) or *Neuron to Astrocyte Lactate Shuttle* (NALS) hypotheses, both of which are supported by extensive, but indirect, experimental evidence. This work explores the conditions favouring development of ANLS or NALS phenomenon on the basis of a model that can simulate both by employing the two parameter sets proposed by Simpson et al. (J Cereb. Blood Flow Metab., 27:1766, 2007) and Mangia et al. (J of Neurochemistry, 109:55, 2009). As most mathematical models governing brain metabolism processes, this model is multi-scale in character due to the wide range of time scales characterizing its dynamics. Therefore, we utilize the *Computational Singular Perturbation* (CSP) algorithm, which has been used extensively in multi-scale systems of reactive flows and biological systems, to identify components of the system that (i) generate the characteristic time scale and the fast/slow dynamics, (ii) participate to the expressions that approximate the surfaces of equilibria that develop in phase space and (iii) control the evolution of the process within the established surfaces of equilibria. It is shown that a decisive factor on whether the ANLS or NALS configuration will develop during neuronal activation is whether the lactate transport between astrocytes and interstitium contributes to the fast dynamics or not. When it does, lactate is mainly generated in astrocytes and the ANLS hypothesis is realised, while when it doesn’t, lactate is mainly generated in neurons and the NALS hypothesis is realised. This scenario was tested in exercise conditions.

## Introduction

The human brain uses glucose as its main source of energy. Even though the adult brain accounts for ∼2% of the body weight, it consumes ∼20% of glucose-derived energy. Brain neurons have the highest energy demand [1] and the brain becomes dependent on glucose for proper functioning.

Glucose is metabolized to pyruvate in the presence oxygen, in both astrocytes and neurons. Pyruvate can follow two different pathways: oxidative or aerobic (producing energy in the mitochondria, in the presence of oxygen) and non-oxidative or anaerobic (producing energy and lactate in the cytosol, before the involvement of oxygen). Even though brain metabolism has been the subject of interest and research for decades, there is still a lot of discussion on to what extent energy derived from glucose is consumed by the astrocytes and the neurons themselves, and how much is shuttled between astrocytes and neurons in the form of lactate [2]. Two different hypotheses were introduced for the shuttling of lactate; one advocating the flow of lactate from astrocytes to neurons and another one advocating the flow of lactate from neurons to astrocytes. These two hypotheses are supported by experimental data, which are indirect “because current technology does not have adequate spatiotemporal resolution to quantify metabolic activity in single cells in vivo” [3].

The astrocyte-to-neuron lactate shuttle (ANLS) was proposed on the basis of glutamate-evoked increases in glucose utilization and lactate release by cultured astrocytes [4, 5]. According to ANLS, the neuronal activity triggers a glucose uptake in astrocytes, which leads to large increases in production of lactate. Lactate is then released to the extracellular space and transports to nearby neurons, where it is used as substrate for energy production (for detailed review see Ref. [5]). The validity of the ANLS hypothesis has been supported by experimental evidence over the years; many of them can be found in Refs. [6, 7].

On the other hand, the neuron-to-astrocyte lactate shuttle (NALS) states that lactate is generated by neurons and taken up by astrocytes during activation. Due to the high energy demands in neuronal activation, neurons take up glucose and transfer lactate to astrocytes, since the neuronal glucose transporter, GLUT3, appears to have higher transport rate compared with the astrocytic glucose transporter, GLUT1 [8–12]. Similarly to the ANLS hypothesis, there is experimental evidence that supports the NALS hypothesis [13–15].

In the context of the ANLS hypothesis, neurons are unable to increase their glycolytic activity in response to neuronal activation [16–18]. Therefore, enhanced glycolysis due to neuronal activation can take place only in astrocytes. On the other hand, in the context of the NALS hypothesis, neuronal activation enhances glycolysis in neurons [19–21].

A large portion of the scientific community is currently split between the validity of either the ANLS or NALS hypothesis, while another portion advocates that both hypotheses can be valid depending on the operating conditions. There is a large body of literature advocating the validity of ANLS [6, 22–32], as this is supported by indirect experimental findings [4, 18, 32–44]. The main criticism of the ANLS hypothesis stems from the NALS supporters [8, 9, 45–54], and the related indirect experimental evidence [10, 13–15, 55–64]. On the other hand, recent findings suggest that perhaps brain responds differently to different environmental challenges, depending on the availability of the fuel source. For example, it was shown that under inadequate supply of glucose, alternative substrates contribute to brain energetics [7, 11], such as lactate and/or ketones during exercise, hypoxia, hypoglycemia or intense brain activity [65, 66]. Therefore, under different brain environmental conditions either the ANLS or NALS hypothesis can be valid. The identification of the conditions under which ANLS or NALS manifests is one of the objectives of this manuscript.

The very beginning of the brain chemistry can be traced back to 1884 [67] but early evidence on metabolism in brain tissue can be found in Himwich’s early work [68] dated in 1951. Since then, the brain energy metabolism network has been investigated extensively mainly through in vitro and in vivo experiments and measurements on rats (e.g., Refs. [34, 69–76]) and humans (e.g., Refs. [55, 70, 77–79]).

On the other hand, the development of mathematical models that aim to simulate the metabolic interactions between metabolites into and among the various cerebral compartments has been flourishing very recently. Aubert et al. were among the first to develop mathematical brain metabolism models in order to investigate the connections between electrical activity, energy metabolism and hemodynamics [80, 81]. These models served as baselines for the development of more detailed ones with various investigation objectives like: the ANLS hypothesis [82], the initial rapid decrement in the extracellular lactate concentration [83], the blood oxygenation level-dependent (BOLD) signal in functional MRI (fMRI) [84], the inclusion of glycogen dynamics in astrocytes [85]. The model proposed by Simpson et al. [9] was developed with the purpose of addressing the role of transport in cerebral metabolism and the limitations of the previously developed models related to the concentrations and kinetic properties of the glucose transporters (GLUTs) and monocarboxylate transporters (MCTs) in the brain. With this work, Simpson et al. provided the setup for both the ANLS and the NALS hypotheses. Although, this model was criticized for its simplisity, its increased accuracy compared to in vivo rodent data lead to its utilization (with small parameters’ adjustments) for studying in vivo data from humans at high field functional magnetic resonance spectroscopy (fMRS) by Mangia et al. [10].

Combining the models proposed in [9, 10, 81, 82], Di Nuzzo et al. [8] introduced a new model to investigate the effects of cellular transport, metabolic capacity, specialization for energy use and metabolite trafficking during normal and enhanced cerebral activity. Aiming on the steady state conditions of the metabolic pathways and their relation to neuronal activation, Occhipinti et al. initially proposed a model [86] and used statistical tools to examine the reaction fluxes and the transport rates. Later this model was revised in [87] in order to investigate the preferred energy substrate (i.e., glucose or lactate) in neurons at high activity. They further investigated the reasons of production of *γ*-aminobutyric acid (GABA), by revising accordingly their latest model in [88]. By modifying and extending the models of Occhipinti et al. [87, 88] for glutamatergic neurons and astrocyte-GABAergic neuron cellular complex, respectively, Calvetti at al. [89] proposed a very detailed steady state model comprising of three cells, each one subdivided into cytosol and mitochondria, where astrocytes, glutamatergic, and GABAergic neurons interact through a common extracellular space (ECS). Finally, the models proposed in [82, 84] were used recently as baseline for the development of a more sophisticated model by Jolivet et al. [22], in order to investigate the ANLS hypothesis and its connection to cerebral blood flow.

Sensitivity analysis and flux analysis are the standard and widely used techniques for the investigation of such complex models. The former technique quantifies the effect of a perturbation in the model’s parameters to the evolution of the system. It is often utilized to obtain insights into the metabolic properties of the system by examining the derivative of fluxes with respect to each model parameters [8, 85], but also to quantify the relative contribution of input parameters on the features of an output attribute, such as BOLD signal [90, 91]. On the other hand, flux balance analysis targets on finding the physiological feasible configuration of reaction fluxes and transport rates capable on maintaining a steady state. It is often utilized to verify the hypotheses on the role of different pathways related to a brain function that have been proposed and to predict the activation levels of pathways at different inputs according to the initial hypotheses [86, 92]. Flux balance analysis can be applied in a mathematical model even when only the stoichiometry is known, and therefore it has been widely used and extended in both deterministic [93, 94] and stochastic frameworks [87, 95, 96]. Flux balance analysis is indeed useful in the interrogation of biochemical systems, but its value is limited since it can lead to conclusions that are only global in character. In addition, sensitivity coefficients (determined through conventional local sensitivity analysis) can be misleading, since a parameter can be related to insignificant sensitivity coefficient and yet be important [97]. Moreover, even in the early 80s works it was acknowledged that there are functional connections between the sensitivity coefficients that they cannot be identified by their mere inspection, thus, additional treatment of the local sensitivity coefficients was proposed [98]. As such, none of these methods can provide details on the temporal evolution of the set of biochemical reactions and metabolites that are most influential on the dynamics and progress of the system [94]. Moreover, they cannot identify the chemical reactions that control the evolution of a metabolite or a variable in general. Such identifications are of paramount importance since they can facilitate the associated physical understanding and eventually can lead to the development of efficient drugs.

Here, the *Computational Singular Perturbation* (CSP) algorithm will be employed for the analysis of a brain lactate metabolism model. CSP is an algorithmic method for asymptotic analysis developed in the late 1980s by Lam and Goussis [99–101]. It exploits the fact that systems describing the time evolution of biological kinetics models, are driven by processes characterized by a wide range of time scales. In such systems, the processes characterized by the fastest time scales become quickly equilibrated (exhausted). The system is thus confined to evolve within the related surfaces of equilibria that emerge in phase space and is driven by the processes that are characterized by the slow time scales. CSP can provide both mathematical expressions that approximate the generated surfaces of equilibria and the simplified system that approximates the slow evolution along these surfaces. CSP was initially developed to treat large and complex chemical kinetic mechanisms in the context of reacting flows [100, 102], but was later employed for the analysis of other physical problems, like computational mechanics [103], atmospheric science [104], etc., including biological systems [105–108] and pharmacokinetics [109, 110]. CSP exhibits many advantages compared to other similar approaches. Firstly, it is fully algorithmic, requiring no input from the investigator, apart from the detailed model and the accuracy that the simplified model is required to provide. In addition, the CSP algorithm can identify the physical processes in the model that contribute to the emergence of the surfaces of equilibria, those that control the slow (long-term) evolution of the system and those that are responsible for the development of the fast and slow time scales. Finally, CSP can identify the variables whose evolution is characterised by the fast or slow time scales. This understanding allows the investigator to identify the processes that can control not only the slow evolution of the whole system, but also the evolution of each individual metabolite; e.g., see the applications in pharmacokinetics [109, 110].

The CSP algorithm refers to the *Geometric Singular Perturbation* (GSP) theory, which introduced slow invariant manifolds (low dimensional surfaces on which trajectories evolve according to the slow time scales) and the fast fibers (along which trajectories approach the slow manifold according to the fast time scales) [111–113]. At each point on the manifold, CSP provides basis vectors that span the fast and slow subdomains of the tangent space. These vectors are provided by two iterative procedures; one that improves the accuracy of the basis vectors that are tangent to the manifold and one that improves the accuracy of the basis vectors aligned with the fast fibers. Considering the small parameter *ϵ*, equal to the ratio of the characteristic fast and slow time scales, each of these iterations increases the accuracy of the CSP basis vectors by O(ϵ) [114–117]. With this set of basis vectors being available, the slow manifold is approximated by the relations that result from the negligible projection of the vector field along the fast basis vectors and the system that governs the flow on the manifold is approximated by the projection of the vector field along the slow basis vectors [100]. Due to the multi-scale character and the complexity of the models that are currently of interest, approaches based on the GSP theory are becoming popular in the field of biology [118–122]. The major advantage of CSP is that is algorithmic and does not require the mathematical model to be cast in a non-dimensional form [105–110].

In the current study, CSP and its algorithmic tools are employed in order to analyse brain lactate metabolism, using the model proposed by Simpson et al. [9], as modified by Mangia et al. [10]. This model was selected for the following reasons. Firstly, it is a fairly simple model, thus, an ideal candidate for the demonstration purpose of the proposed method (i.e., CSP and its tools). Secondly, the model of Mangia et al. [10] provides a basis for both the ANLS and the NALS hypotheses. Therefore, both hypotheses can be examined, in view of the same mechanism, which can lead to a fair comparison of the results. Thirdly, it is among the very few models that was fitted against human in vivo data [10, 28, 84], in contrast to the majority of the models that are calibrated against in vitro human data [8, 80–84, 88, 89] or in vivo rat data [9, 28, 85].

In the following, results obtained from the CSP analysis of a simple brain lactate metabolism model will be reported. By investigating the fast and slow dynamics of the model, the couplings among the glucose and lactate pathways are revealed in the context of both the ANLS and the NALS hypotheses. In addition, the rate limiting steps in each of the two pathways are identified. These findings are crucial in order to understand the mechanism for lactate shuttling in the brain, and for the identification of the most conducive point in the pathways for the control of the process. These findings will be demonstrated in normal and exercise conditions of neuronal activation.

## Methods

### The computational model

**Fig 1:**
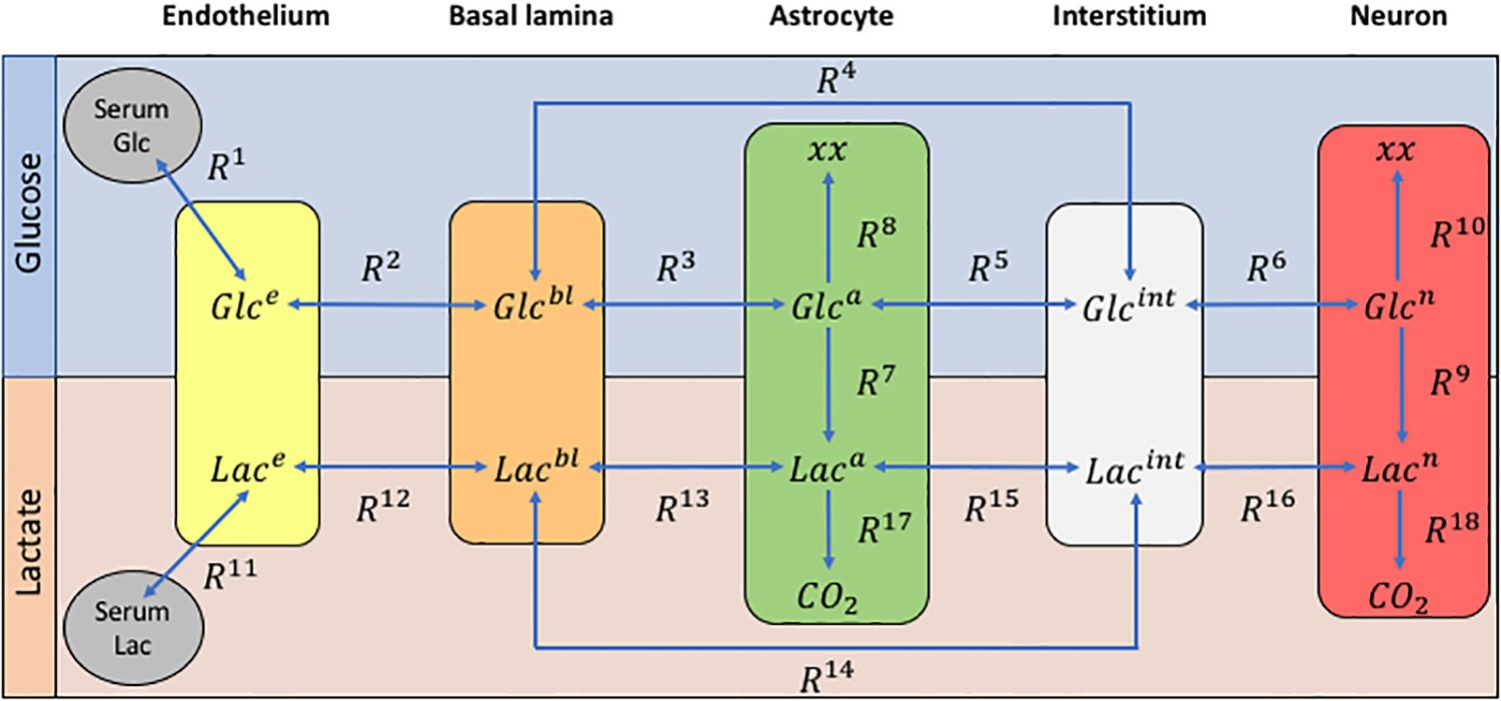
The 5-compartmental model introduced in [9]. The initial quantity of *Glc* and *Lac* is provided to the system through serum and then *Glc*^*i*^ and *Lac*^*i*^ transports, diffuses or metabolizes through the various compartments, (*i* = *e*, *bl*, *a*, *int*, *n*), by the 12 reversible and the 6 irreversible reactions.

The brain lactate metabolic network is analyzed by utilizing the model introduced in *Simpson et al*. [9], as modified by Mangia et al. [10], which simulates the kinetic behavior of glucose and lactate among the cerebral compartments. As shown in Fig 1 the model consists of 5 compartments: the endothelium (*e*), the basal lamina (*bl*), the astrocyte (*a*), the interstitium (*int*) and the neuron (*n*). Glucose (*Glc*) and lactate (*Lac*), which are the only chemical species that are accounted for in the model, are oxidized in astrocytes and neurons in order to produce energy. The transport or diffusion of these two substances from one compartment to another is denoted as a reaction. As shown in Fig 1, there are 12 reversible and 6 irreversible reactions in the model; see S1 Text for a detailed description of the model. At this point it suffices to state that the interactions of *Glc* and *Lac* through the various compartments is governed by a 10-dimensional system of Ordinary Differential Equations (ODEs):

$$\begin{array}{c} \frac{dy}{dt}=\sum_{k=1}^{K}S_{k}R^{k}\left(y\right)=g(y) \end{array}$$ (1)

where **y** is the *N*-dim. column vector containing the concentrations of species *Glc*^*i*^ and *Lac*^*i*^ at the five compartments (*N* = 2 × 5 = 10), **S**_*k*_ denotes the *N*-dim. stoichiometric vector of the *K* unidirectional reactions and *R*^*k*^ is the related reaction rate. In order to assess the influence of the two directions in a reversible reaction, in the analysis that follows the contribution of a reversible reaction **S**_*i*_*R*^*i*^ will be considered as a contribution of two unidirectional reactions, $S_{R}^{i,f}$ and $S_{R}^{i,b}$, respectively (*K* = 12 × 2 + 6 = 30). The system will be analysed for normal and exercise conditions, which are distinguished by the level of lactate in the serum; *Glc*^*s*^ = 5.5 mmol/L and *Lac*^*s*^ = 1 mmol/L in the case of normal conditions and *Glc*^*s*^ = 5.5 mmol/L and *Lac*^*s*^ = 20 mmol/L in the case of exercise conditions [123, 124].

### Computational singular perturbation (CSP) method and algorithmic tools

The Computational Singular Perturbation (CSP) method relies on the multi-scale character of the system under investigation and leads to an algorithm that delivers everything the traditional singular perturbation technique does. As the system evolves, CSP identifies the subspaces in phase space, in which the fast and slow dynamics act. The components of the model that tend to drive the system along the fast directions equilibrate, generating thus constraints in which the system is bound to evolve. This evolution is driven by components that act along the slow directions. With the fast and slow subspaces provided by CSP at each point in time, the components of the model that are mainly responsible for the generated constraints and for driving the system are easily identified.

According to the CSP approach, the vector field **g**(**y**) is resolved in *N* modes, so that Eq (1) is cast in the form [100, 125]:

$$\begin{array}{c} \frac{dy}{dt}=g\left(y\right)=\sum_{n=1}^{N}a_{n}\left(y\right)f^{n}\left(y\right)\;f^{n}\left(y\right)=b^{n}\left(y\right)\cdot g\left(y\right)=\sum_{k=1}^{K}(b^{n}\left(y\right)\cdot S_{k})R^{k}\left(y\right) \end{array}$$ (2)

where **a**_*n*_ is the *N*-dim. CSP column basis vector of the *n*-th mode, **b**^*n*^ is the *N*-dim. dual row vector that satisfies the orthogonality conditions $b^{i}\cdot a_{j}=\delta_{j}^{i}$ and *f*^*n*^ is the related amplitude (set positive by properly adjusting the sign of the *N*-dim. row vectors **b**^*n*^) [99, 100]. Each mode **a**_*n*_ *f*^*n*^ relates to a distinct time scale, say *τ*_*n*_; see S2 Text. The amplitude *f*^*n*^ of the *n*-th mode provides a measure of the projection of the vector field **g**(**y**) on the CSP vector **a**_*n*_. Therefore, when the system in Eq (2) exhibits *M* time scales that are (i) of dissipative nature, i.e. the components of the system that generate them tend to drive the system towards a fixed point and (ii) much faster than the rest, the following reduced model is obtained:

$$\begin{array}{c} f^{r}\left(y\right)\approx 0\;\left(r=1,\ldots ,M\right)\;\;\frac{dy}{dt}\approx \sum_{s=M+1}^{N}a_{s}\left(y\right)f^{s}\left(y\right) \end{array}$$ (3)

when these *M* fast time scales become exhausted. Interested in leading order accuracy, the CSP vectors **a**_*i*_ and **b**^*i*^ (*i* = 1, …, *N*) can be approximated by the right and left, respectively, eigenvectors of the *N* × *N*-dim. Jacobian **J** of **g**(**y**); i.e., **a**_*i*_ = *α*_*i*_ and **b**^*i*^ = *β*^*i*^ [100, 125, 126]. The first relation in Eq (3) is an *M*-dim. system of algebraic equations and defines the manifold M (a low dimensional surface in phase-space, where the system is confined to evolve), while the second relation is an *N*-dim. system of ODEs that governs the slow evolution of the system on this manifold. In the following, the dependency of *R*^*k*^, **g**, ***α***, etc. from **y** will be omitted for simplicity.

The *M* constraints in Eq (3) *f*^*r*^ = (***β***^*r*^ ⋅ **S**_1_)*R*^1^ + … + (***β***^*r*^ ⋅ **S**_*K*_)*R*^*K*^ ≈ 0 (*r* = 1, …, *M*) are the result of significant cancellations among some of the additive terms (***β***^*r*^ ⋅ **S**_*k*_)*R*^*k*^ (*k* = 1, …, *K*). The reactions that contribute significantly to the formation of each of the *M* constraints are identified by the *Amplitude Participation Index* (*API*):

$$\begin{array}{c} P_{k}^{r}=\frac{\left(\beta^{r}S_{k}\right)R^{k}}{\sum_{i=1}^{K}\left|\left(\beta^{r}S_{i}\right)R^{i}\right|}.\;\;\left(k=1,\ldots ,K\right) \end{array}$$ (4)

where by definition $\sum_{k=1}^{K}\left|P_{k}^{r}\right|=1$, [99, 127, 128]. $P_{k}^{r}$ provides a measure of the contribution of the *k*-th reaction to the cancellations among the additive terms in *f*^*r*^ ≈ 0 and can be either positive or negative, the sum of positive and negative terms equaling 0.5, by definition.

The formation of the *M* constraints and the dynamics of the slow system in Eq (3) are characterized by the *M* fastest time scales and by the fastest of the *N* − *M* slow ones, respectively. These time scales are approximated by the inverse of the eigenvalues of the Jacobian **J**, *τ*_*n*_ = |λ_*n*_|^−1^ (*n* = 1, …, *N*). The reactions that contribute significantly to the generation of these time scales are identified by the *Time scale Participation Index* (*TPI*):

$$\begin{array}{c} J_{k}^{n}=\frac{c_{k}^{n}}{\sum_{i=1}^{K}\left|c_{i}^{n}\right|}\;\;\left(k=1,\ldots ,K\right) \end{array}$$ (5)

where $\lambda_{n}=c_{1}^{n}+\ldots +c_{K}^{n}$ and by definition $\sum_{k=1}^{K}\left|J_{k}^{n}\right|=1$ [127, 129, 130]. $c_{k}^{n}$ denotes the contribution of the *k*-th reaction to the *n*-th eigenvalue and can be calculated as $c_{k}^{n}=\beta^{n}\nabla (S_{k}R^{k})\alpha_{n}$, where $\sum_{k=1}^{K}\nabla (S_{k}R^{k})$ is the Jacobian **J**; see S2 Text for details. $c_{k}^{n}$ can be either positive or negative and therefore, when $J_{k}^{n}$ is positive (negative), it implies that the *k*-th reaction contributes to an explosive (dissipative) character of the *n*-th time scale *τ*_*n*_. By definition, explosive (dissipative) time scales relate to the components of the system that tend to drive it away from (towards) a fixed point [99, 100].

Each chemical species associates differently to each exhausted CSP mode, e.g., a chemical species can relate mostly to the *m*-th CSP mode (*m* = 1, …, *M*) and much less to the rest. The relation of the *m*-th CSP mode to the various chemical species is assessed by the *CSP Pointer* (*Po*):

$$\begin{array}{c} D^{m}=diag[\alpha_{m}\beta^{m}]=[\alpha_{m}^{1}\beta_{1}^{m},\alpha_{m}^{2}\beta_{2}^{m},\ldots ,\alpha_{m}^{N}\beta_{N}^{m}]\;\;\left(m=1,\ldots ,M\right) \end{array}$$ (6)

where, due to the orthogonality condition $\beta^{i}\cdot \alpha_{j}=\delta_{j}^{i}$, the sum of all *N* elements of **D**^*m*^ equals unity; i.e., $\sum_{i=1}^{N}\alpha_{m}^{i}\beta_{i}^{m}=1$ [99, 128, 131, 132]. Values of $\alpha_{m}^{i}\beta_{i}^{m}$ close to unity indicate that the *i*-th variable is strongly connected to *m*-th CSP mode and the corresponding time scale. Chemical species with *Po* values close to unity are potentially in *Quasi Steady-State* (QSS) [132].

The reactions that contribute the most to the evolution of the system within the *M* constraints, according to the *N*-dim. system of ODEs in Eq (3), are identified by the *slow Importance Index* (*II*):

$$\begin{array}{c} I_{k}^{n}=\frac{\sum_{s=M+1}^{N}\alpha_{s}^{n}\left(\beta^{s}\cdot S_{k}\right)R^{k}}{\sum_{j=1}^{K}\left|\sum_{s=M+1}^{N}\alpha_{s}^{n}\left(\beta^{s}\cdot S_{j}\right)R^{j}\right|}\;\;\left(n=1,\ldots ,N,\;\;k=1,\ldots ,K\right) \end{array}$$ (7)

where by definition, $\sum_{k=1}^{K}\left|I_{k}^{i}\right|=1$ [99, 127, 133]. $I_{k}^{n}$ provides a measure of the relative importance of the *k*-th reaction to the production (when positive) or consumption (when negative) of the *n*-th chemical species [99, 128, 131, 134]. A detailed description of the CSP methodology is available at S2 Text.

## Results

### The dynamics of the system

The evolution of glucose and lactate concentrations in the various cerebral compartments is simulated during neuronal activation by the model described briefly in the previous section and in detail in S1 Text. The variations of the concentrations of metabolites during neuronal activation (electrical and chemical excitability of neurons) have been recorded experimentally by fMRS [10]. It was shown that neuronal activation begins (ends) with a rapid increase (decrease) in lactate and a decrease (increase) in glucose in the human cortex. In order for the model employed to reproduce this response of the concentrations, the parameters in the model were properly adjusted in the periods of neural activation. In particular, the system was allowed to rest on its baseline steady-state conditions for the first 1000 *s* and then the parameters associated with lactate (glucose) utilization were adjusted in order to generate increased (decreased) levels of lactate (glucose) during the first period of neuronal activation *P*_1_ (1020 *s* < *t* < 1240 *s*). These adjustments were removed at the end of *P*_1_ and the system was let to evolve towards the next period, *P*_2_ (1320 *s* < *t* < 1540 *s*). As in *P*_1_, adjustments in the parameters were introduced throughout *P*_2_ in order to produce decreased (increased) lactate (glucose) levels. After the completion of *P*_2_, the adjustments were removed and the system was allowed to reach steady state conditions. For a detailed explanation of the parameter adjustments during *P*_1_ and *P*_2_ see Ref. [10].

#### Multi-scale character and modal decomposition

The multi-scale character of the model is established by wide gaps among the fast and slow time scales *τ*_*n*_ and by the ensuing negligible amplitude of the fast modes *f*^*n*^.

**Fig 2:**
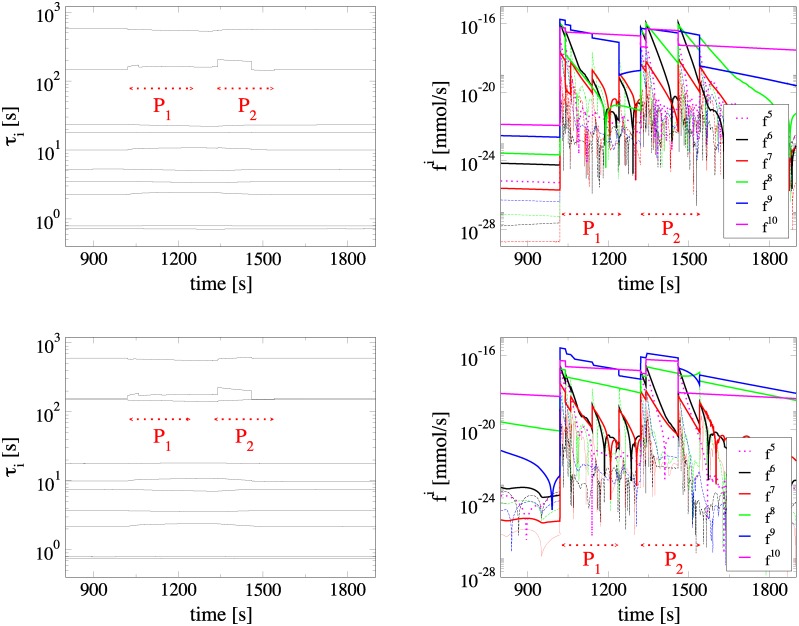
The timescales and the amplitudes. Evolution of the time scales *τ*_*i*_ (left) and the amplitudes *f*^*i*^ (right) of the CSP modes during neuronal activation, for the ANLS (top) and NALS (bottom) cases. *P*_1_ and *P*_2_ represent the periods of neuronal activation that are examined in detail. The dotted and solid lines in the figures on the right denote the first 5 and the last 5 amplitudes *f*^*i*^, respectively.

There are *N* = 10 variables in the model employed, so that according to Eq (2) there are 10 amplitudes, which are related to 10 time scales. Fig 2 displays the evolution of the *N* time scales *τ*_*n*_ that characterise the dynamics of the metabolic network under consideration and the amplitudes *f*^*n*^ of the *N* CSP modes, for both ANLS (top) and NALS (bottom) cases. The period considered includes the periods *P*_1_ and *P*_2_ of neuronal activation, as indicated in the figure. The rapid “step” deviations either in time scale or in amplitude evolution relate to the parameter adjustments during *P*_1_ and *P*_2_. These adjustments have a negligible influence in the time scales, but a significant one in the amplitudes.

In particular, in the left part of Fig 2 it is shown that in both the ANLS and NALS cases the time scale spectrum extends from *O*(1) to *O*(10^3^) s. These time scales are all dissipative (the related eigenvalues are all real and negative), so they are generated by processes that tend to drive the system towards a fixed point. There is a general agreement in the evolution of the time scales in the ANLS and NALS cases. The largest deviations are recorded in the 5th and 8th time scales: *τ*_5_ is somewhat slower in the NALS case, while *τ*_8_ is much slower. The most notable difference in the two cases is that the largest time scale gap is encountered between the 8th and the 9th scales in the ANLS case and between the 7th and the 8th scales in the NALS case. This finding suggests that there is one more constraint established in the ANLS case.

The corresponding amplitudes *f*^*i*^ of the CSP modes are displayed in the right part of Fig 2, where the 5 first modes are denoted with dotted lines and the last 5 with solid ones. Before the neuronal activation (*t* < 1020 *s*), where the system has already attained its steady state conditions, the two largest amplitudes are *O*(10^−23^) and *O*(10^−22^) and relate to the slowest 9th and 10th CSP modes respectively; indicating that all CSP modes have been exhausted (i.e., all 10 modes are inactive). As the neuronal activation manifests itself at *t* = 1020 *s*, all amplitudes increase. The largest amplitudes during the neuronal activation periods P_1_ and P_2_ in both ANLS and NALS cases are those of the two slowest 9th and 10th CSP modes, reaching *O*(10^−16^), followed by the amplitude of the 8th mode. In the ANLS case, the amplitude of the 8th CSP mode relaxes very quickly after its rapid increase at the start of neuronal activation. In contrast, in the NALS case the amplitude of the 8th CSP mode attains a relatively large value throughout the period of neuronal activation. This is in agreement to the finding that the largest fast/slow time scale gap is encountered between the 8th and the 9th time scales in the ANLS case and between the 7th and the 8th time scales in the ANLS case; i.e., Fig 2 shows that during neuronal activation only the amplitudes of the slow modes are large. After the end of P_2_, all amplitudes decay according to the related time scale as the system returns to its steady state; the slowest decay exhibited by the amplitude of the slowest 10th mode.

Our study will focus on the periods *P*_1_ and *P*_2_ of neuronal activation, which are displayed with dashed red arrows in both the left and right panels of Fig 2. Period *P*_1_ relates mostly to the glycolytic uptake in astrocytes, while period *P*_2_ relates to the *Lac* oxidation in both neurons and astrocytes [10]. Both periods display quite similar dynamical behavior. In particular, in both the ANLS and NALS cases the amplitudes of all modes grow at the start of the two activation periods, but the majority of them relax very quickly. In the ANLS case the number of exhausted time scales is *M* = 8 for the largest part of *P*_1_ and *P*_2_. Therefore, the 9th and 10th CSP modes are considered to be the *slow* ones (i.e., the ones that drive the system in *P*_1_ and *P*_2_), while the 1st to the 8th are the exhausted *fast* ones (i.e., their negligible amplitudes define the confines in which the system evolves). In contrast, in the NALS case, the number of exhausted time scales is *M* = 7 for the same periods. Now, the 8th, 9th and 10th CSP modes are considered *slow*, while the 1st to the 7th modes are *fast*.

In the following, the CSP diagnostic tools discussed previously are utilized in order to acquire physical understanding and derive conclusions about the evolution of the system during neuronal activation. Interested in the long-term evolution of the system, the analysis will focus on the slow dynamics. However, in order to understand the influence of the surface of equilibria, which is generated by the *M* constraints, on the behavior of the system, a brief presentation of the established constraints is presented first. These constraints involve the most intense components of the system, the equilibration of which allows the less intense ones to drive the system.

#### Fast dynamics and established constraints

The fast dynamics of the system is responsible for the constraints that are established when the *fast* CSP modes become exhausted (*f*^*r*^ ≈ 0, *r* = 1, *M*). As previously stated, during neuronal activation, the number of exhausted modes is identified to be *M* = 8 and *M* = 7 in the ANLS and NALS cases, respectively. These *fastest* CSP modes were analysed with the CSP tools and the resulting diagnostics are displayed in Table 1 for the ANLS case and in Table 2 for the NALS case (the diagnostics of the non-exhausted 8th mode in the NALS case are included in Table 2 for comparison with the exhausted 8th mode in the ANLS case). The results displayed in the two Tables were computed at *t* = 1100 *s*, which belongs to *P*_1_. These diagnostics are indicative of those throughout *P*_1_ and *P*_2_; only 1-2% differences in the CSP diagnostics are recorded. Along with the Pointer *Po*, the Amplitude Participation Index *API* and the Time scale Participation Index *TPI*, the product **b**^*m*^⋅**S**_*k*_ is displayed in Tables 1 and 2. When $b^{m}\cdot S_{k}=O\left(1\right)$, the stoichiometric vector of the *k*-th reaction has a significant component along **a**_*m*_. Since **a**_*m*_ is a *fast* CSP basis vector, $b^{m}\cdot S_{k}=O\left(1\right)$ indicates that the *k*-th reaction is a fast one and can potentially contribute to the fast dynamics.

**Table 1:** CSP diagnostic tools for the 8 exhausted modes in the ANLS case.

Mode	*Po*		*API*		b^*m*^ ⋅ S_*k*_	*TPI*	
1	*Glc*^*bl*^	0.915	4f	-30.5%	-1.315	4f	-68.7%
	*Glc*^*int*^	0.057	4b	25.2%	1.315	2b	-18.7%
			2f	16.5%	-1.392	4b	-4.9%
			2b	-10.2%	-1.392		
2	*Lac*^*bl*^	0.890	14f	24.9%	1.510	14f	-75.4%
	*Lac*^*int*^	0.108	14b	-24.5%	-1.510	13f	-10.5%
			16f	-15.0%	-0.146	14b	-7.6%
			16b	14.5%	0.146		
			15f	5.6%	0.139		
			15b	-5.1%	-0.139		
3	*Lac*^*int*^	0.697	16f	33.8%	0.694	15b	-30.8%
	*Lac*^*a*^	0.113	16b	-32.4%	-0.694	16f	-25.9%
	*Lac*^*n*^	0.097	15f	-14.1%	-0.742	16b	-21.2%
			15b	12.7%	0.742	15f	-8.8%
4	*Glc*^*e*^	0.693	1f	18.9%	1.276	1b	-34.5%
	*Glc*^*int*^	0.275	1b	-13.7%	-1.276	2f	-33.7%
			2f	-13.4%	-1.243	6f	-11.2%
			6f	10.9%	0.340	4b	-8.2%
			6b	-10.6%	-0.340	5b	-7.4%
			2b	8.3%	1.243		
			4f	-7.0%	-0.328		
			5b	6.2%	0.348		
			4b	5.8%	0.328		
			5f	-5.1%	-0.348		
5	*Glc*^*int*^	0.490	6f	-19.4%	-0.506	6f	-26.4%
	*Glc*^*e*^	0.273	6b	19.0%	0.506	5b	-18.2%
	*Glc*^*a*^	0.083	1f	14.7%	0.791	1b	-16.8%
	*Glc*^*n*^	0.082	5b	-11.5%	-0.542	6b	-7.3%
	*Glc*^*bl*^	0.072	1b	-10.6%	-0.791	4f	-7.2%
			5f	9.5%	0.542	5f	-6.4%
			4f	-3.4%	-0.129		
			4b	2.9%	0.129		
6	*Lac*^*a*^	0.573	16f	31.1%	0.547	16b	-41.4%
	*Lac*^*n*^	0.423	16b	-29.9%	-0.547	15f	-25.4%
			15f	14.5%	0.638	16f	22.0%
			15b	-13.1%	-0.638	15b	4.9%
			7	-3.3%	-1.410		
			18	-1.6%	-0.485		
			17	1.6%	0.700		
7	*Lac*^*e*^	0.992	16f	-16.5%	-0.010	11b	-46.7%
			16b	15.9%	0.010	12f	-45.5%
			12b	10.7%	0.974		
			11b	-10.4%	-0.999		
			12f	-10.1%	-0.974		
			11f	9.9%	0.999		
8	*Glc*^*a*^	0.623	6f	25.4%	0.639	5f	-51.3%
	*Glc*^*n*^	0.373	6b	-24.8%	-0.639	6b	-34.5%
			5b	-19.7%	-0.882	5b	6.2%
			5f	16.2%	0.882	6f	-1.9%
			7	4.1%	0.962	2b	-0.1%
			1f	1.4%	0.073		
			1b	-1.0%	-0.073		

ANLS: CSP diagnostic tools (*Po*, *API*, **b**^*m*^ ⋅ **S**_*k*_ and *TPI*) for the 8 exhausted modes during neuronal activation; *t* = 1100 *s*. Only the largest contributions are presented.

**Table 2:** CSP diagnostic tools for the 7 exhausted and 1 active modes in the NALS case.

Mode	*Po*		*API*		b^*m*^ ⋅ S_*k*_	*TPI*	
1	*Glc*^*bl*^	0.914	4f	33.1%	1.312	4f	-72.0%
	*Glc*^*int*^	0.056	4b	-27.7%	-1.312	2b	-19.0%
			2f	-16.7%	-1.053	4b	-5.2%
			2b	10.9%	1.053		.
2	*Lac*^*bl*^	0.887	14b	-24.2%	-1.326	14f	-75.3%
	*Lac*^*int*^	0.111	14f	24.0%	1.326	13f	-10.3%
			16b	16.4%	0.161	14b	-7.7%
			16f	-16.1%	-0.161		
			15b	-5.0%	-0.154		
			15f	4.8%	0.154		
3	*Lac*^*int*^	0.700	16b	35.2%	0.588	15b	-29.4%
	*Lac*^*a*^	0.109	16f	-34.7%	-0.588	16f	-27.5%
	*Lac*^*n*^	0.094	15b	-12.1%	-0.633	16b	-20.6%
			15f	11.6%	0.633	15f	-8.8%
4	*Glc*^*e*^	0.870	1f	23.4%	1.185	1b	-42.2%
	*Glc*^*int*^	0.115	1b	-17.4%	-1.185	2f	-37.3%
			2f	-15.4%	-1.322	4b	-5.8%
			2b	10.0%	1.322	6f	-4.4%
			4f	-9.2%	-0.297	5b	-0.2%
			6f	8.4%	0.187		
			4b	7.7%	0.297		
			6b	-7.6%	-0.187		
			5b	0.4%	0.163		
			5f	-0.2%	-0.163		
5	*Glc*^*int*^	0.593	6f	36.7%	0.927	6f	-53.6%
	*Glc*^*n*^	0.249	6b	-33.3%	-0.927	6b	-24.3%
	*Glc*^*e*^	0.093	1f	-12.2%	-0.703	1b	-9.0%
	*Glc*^*bl*^	0.064	1b	9.1%	0.703	4f	-4.9%
	*Glc*^*a*^	0.001	4f	2.4%	0.009	5b	-2.3%
			4b	-2.0%	-0.009	5f	-1.3%
			5b	1.3%	0.684		
			5f	-0.7%	-0.684		
6	*Lac*^*a*^	0.604	16b	32.5%	0.426	16b	-39.8%
	*Lac*^*n*^	0.389	16f	-32.0%	-0.426	15f	-27.4%
			15b	13.2%	0.542	16f	20.4%
			15f	-12.7%	-0.542	15b	5.8%
			9	-2.0%	-0.729		
			18	1.5%	0.365		
			17	-1.4%	-0.604		
			7	0.9%	1.204		
7	*Lac*^*e*^	0.992	16b	18.1%	0.001	11b	-46.7%
			16f	-17.8%	-0.001	12f	-45.4%
			12b	10.2%	1.011		
			11b	-10.0%	-0.985		
			12f	-9.8%	-1.011		
			11f	9.7%	0.985		
8	*Glc*^*a*^	0.564	6f	-13.2%	-0.008	5f	-27.3%
	*Glc*^*n*^	0.337	1f	-12.7%	-0.167	6b	-18.5%
			6b	12.0%	0.008	6f	15.6%
			5b	9.8%	1.159	5b	-11.2%
			1b	9.4%	0.167	4b	-6.5%
			2f	-9.0%	-0.494		
			9	7.2%	0.446		
			2b	5.9%	0.494		
			5f	-5.4%	-1.159		
			7	-3.6%	-0.790		

NALS: CSP diagnostic tools (*Po*, *API*, **b**^*m*^ ⋅ **S**_*k*_ and *TPI*) for the 7 exhausted modes during neuronal activation; *t* = 1100 *s*. Only the largest contributions are presented. The CSP data are also displayed for the non-exhausted 8th mode for comparison with the ANLS case.

As stated previously, the *m*-th exhausted CSP mode is distinguished by (i) the reactions that participate in the constraint expressed by the relation *f*^*m*^ ≈ 0 (identified by the *API* index, Eq (4)), (ii) the reactions that generate the time scale *τ*_*m*_ that characterizes the formation of this constraint (identified by the *TPI* index, Eq (5)) and (iii) the variables (concentrations) that relate the most to this mode (identified by the *Po* index, Eq (6)). These CSP diagnostics for the exhausted modes are displayed in Tables 1 and 2.

The main observation from Tables 1 and 2 is that among the eight fastest modes 4 relate to the Glucose path and 4 relate to the Lactate path; i.e., the 1st, 4th, 5th and 8th modes relate to variables and reactions along the Glucose path (Glc-path modes) and the 2nd, 3rd, 6th and 7th ones relate to variables along the Lactate path (Lac-path modes). In particular,

1. The pointed by *Po* variables of the Glc-path modes (Lac-path modes), relate only to *Glc* (*Lac*).
2. The reactants of the reactions identified by *TPI* to contribute to the development of the four time scales *τ*_1_, *τ*_4_, *τ*_5_, *τ*_8_ that relate to the Glc-path modes (*τ*_2_, *τ*_3_, *τ*_6_, *τ*_7_ that relate to the Lac-path modes), involve only *Glc* (*Lac*).
3. The reactants of the reactions identified by *API* to contribute to the constraints *f*^*m*^ ≈ 0, associated to the four Glc-path modes, involve only *Glc*. To a large extent, a similar situation arises in the four constraints that relate to the Lac-path modes; i.e., the reactants of almost all contributing reactions involve *Lac*. The only exception in this case is that in the constraint related to the 6th Lac-path mode the *Glc*-consuming reaction 7 provides a small contribution in the ANLS case, while the *Glc*-consuming reaction 9 provides a small contribution in the NALS case.

Therefore, considering the couplings of the two paths in the expressions that approximate the manifold M:

1. the Lactate path couples to the Glucose path through the 6th mode; this coupling takes place inside the astrocyte compartment in the ANLS case (reaction 7) and in the neuron compartment in the NALS case (reaction 9) and
2. the Glucose path is decoupled from the Lactate path in both the ANLS and NALS cases.

The conclusion is that a perturbation in the reactions on the Lactate path will not alter the Glucose-related constraints. On the other hand, a perturbation in the reactions on the Glucose path will alter the Lactate constraints; as stated previously, this influence is exercised inside the astrocyte compartment in the ANLS case and in the neuron compartment in the NALS case.

Let us now examine the four constraints that develop along the Glucose path, on the basis of the CSP diagnostics displayed in Tables 1 and 2; i.e., those related to the 1st, 4th, 5th and 8th modes in the ANLS case and those related to the 1st, 4th and 5th modes in the NALS case. The 1st (fastest) mode denotes in both ANLS and NALS cases an equilibration between the 2nd and the 4th reactions:

$$\begin{array}{c} -z_{4}^{1}\left(R^{4f}-R^{4b}\right)+z_{2}^{1}\left(R^{2f}-R^{2b}\right)\approx 0 \end{array}$$ (8)

where $z_{k}^{m}=\left|b^{m}\cdot S_{k}\right|$ and the ordering of the reactions is on the basis of their *API* (largest *API*s first). This constraint refers to *Glc*^*bl*^, which is the pointed by *Po* variable and reactant of the reactions that provide the largest contribution to *τ*_1_; i.e., reactions 4f and 2b, according to the *TPI* values displayed in the first row of Tables 1 and 2. The next fastest mode along the Glucose path is the 4th and, according to *API* data, in both ANLS and NALS cases this mode denotes an equilibration among the 1st, 2nd, 4th, 5th and 6th reactions:

$$\begin{array}{c} z_{1}^{4}\left(R^{1f}-R^{1b}\right)-z_{2}^{4}\left(R^{2f}-R^{2b}\right)+z_{6}^{4}\left(R^{6f}-R^{6b}\right)-z_{4}^{4}\left(R^{4f}-R^{4b}\right)-z_{5}^{4}\left(R^{5f}-R^{5b}\right)\approx 0 \end{array}$$ (9)

According to *Po*, this mode mainly refers to *Glc*^*e*^ and secondarily to *Glc*^*int*^. *Glc*^*e*^ is reactant of reactions 1b and 2f, which provide the largest contributions to *τ*_4_, while *Glc*^*int*^ is reactant of reactions 6f, 4b and 5b, which provide the smaller contributions; see the diagnostics in the second row of Tables 1 and 2. The third fastest mode along the Glucose path is the 5th and denotes the equilibration among the 1st, 4th, 5th and 6th reactions:

$$\begin{array}{c} -z_{6}^{5}\left(R^{6f}-R^{6b}\right)+z_{1}^{5}\left(R^{1f}-R^{1b}\right)+z_{5}^{5}\left(R^{5f}-R^{5b}\right)-z_{4}^{5}\left(R^{4f}-R^{4b}\right)\approx 0 \end{array}$$ (10)

The largest contributions in the cancellations occurring in this constraint originate from the two directions of the 6th reaction; this feature is more pronounced in the NALS case. The contributions of the two directions of the 1st reaction are the next largest and are similar in both cases. The smallest contributions originate from the 4th and 5th reactions; the ones of the 4th reaction being similar in the two cases and those of the 5th reactions becoming negligible in the NALS case. This is the first mode along the Glucose path in which notable differences are encountered in the ANLS and NALS cases; i.e., a decreasing contribution of the 5th reaction and an increasing one of the 6th reaction in the NALS case relative to the ANLS one. This feature, that emerges when switching from the ANLS to the NALS case, is accompanied by the change in the way the Lactate path is coupled to the Glucose path. Specifically, in the ANLS case this coupling takes place in the astrocytes (where the 5th reaction is active) via the 7th reaction, while in the NALS case this coupling takes place in the neurons (where the 6th reaction is active) via the 9th reaction. This mode (5th) refers mainly to *Glc*^*int*^ and secondarily to *Glc*^*e*^ in the ANLS case and to *Glc*^*n*^ in the NALS case. *Glc*^*int*^ is the reactant of reaction 6f that provides the largest contributions to *τ*_5_ in both cases and of reaction 5b that provides a significant contribution in the ANLS case. The last fast mode along the Glucose path is the 8th and emerges only in the context of the ANLS case. This mode denotes the equilibration among the 5th, 6th and 7th reactions:

$$\begin{array}{c} z_{6}^{8}\left(R^{6f}-R^{6b}\right)+z_{5}^{8}\left(R^{5f}-R^{5b}\right)+z_{7}^{8}R^{7}\approx 0 \end{array}$$ (11)

and refers mainly to *Glc*^*a*^ and secondarily to *Glc*^*n*^. *Glc*^*a*^ is the reactant of reaction 5f that provides the largest contribution to *τ*_8_, while *Glc*^*n*^ is the reactant of reaction 6b that provides the next largest contribution. Table 2 shows that in the NALS case the 8th mode is not exhausted (since no large cancellations among the APIs are evident). However, the pointed variables and the reactions contributing to *τ*_8_ are the same. As Fig 2 shows, this mode is not exhausted because *τ*_8_ is significantly slower in the NALS case, compared to the ANLS one.

**Fig 3:**
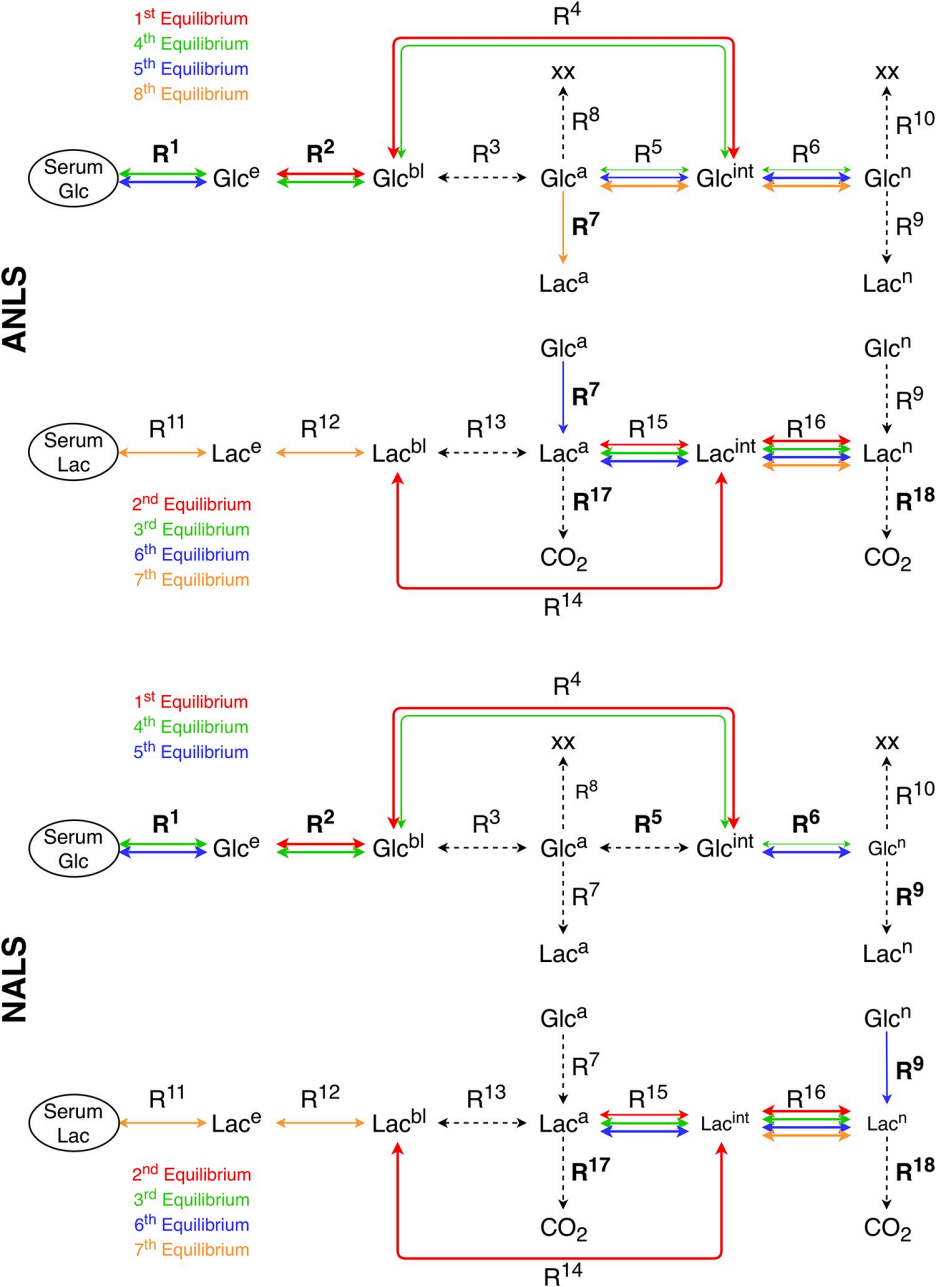
The constraints along the glucose and lactate paths. The constraints that develop along the Glucose and Lactate paths in the ANLS (top) and NALS (bottom) cases. Solid (dashed) arrows indicate reactions exhibiting large (small) APIs. The reactions within these constraints that drive the system (exhibit large IIs) are indicated by bold; see discussion of the slow model.

A schematic representation of these constraints that develop along the Glucose path in the ANLS and NALS cases is shown in Fig 3. Clearly, the *Glc*-related constraints involve all five compartments along the Glucose path and all means of transport and diffusion among them, with the contribution of the 3rd reaction being negligible, since its rate is sufficiently small; see S1 Text.

The four constraints that develop along the Lactate path relate to the 2nd, 3rd, 6th and 7th modes, for both the ANLS and NALS cases. On the basis of the CSP diagnostics displayed in Tables 1 and 2, the following conclusions can be reached. The 2nd mode denotes mainly the equilibration between the forward and backward directions of the 14th reaction, corrected by the net contribution of the 16th and 15th reactions:

$$\begin{array}{c} -z_{14}^{2}\left(R^{14f}-R^{14b}\right)+z_{16}^{2}\left(R^{16f}-R^{16b}\right)-z_{15}^{2}\left(R^{15f}-R^{15b}\right)\approx 0 \end{array}$$ (12)

The mode refers to *Lac*^*bl*^, which is the pointed by *Po* species in both NALS and ANLS cases and is reactant of the reactions that provide the largest contributions to *τ*_2_, 14f and 13f, according to *TPI*. The less pointed *Lac*^*int*^ is reactant of reaction 14b, which provides a small contribution to *τ*_2_. The 3rd mode expresses the equilibration of the 15th and 16th reactions:

$$\begin{array}{c} z_{16}^{3}\left(R^{16f}-R^{16b}\right)-z_{15}^{3}\left(R^{15f}-R^{15b}\right)\approx 0 \end{array}$$ (13)

which, according to *Po*, mainly refers to *Lac*^*int*^ and secondarily to *Lac*^*a*^ and *Lac*^*n*^ in both cases. *Lac*^*int*^ is reactant of reactions 16f and 15b, which provide the largest contributions to *τ*_3_, while *Lac*^*a*^ and *Lac*^*n*^ are reactants of reactions 15f and 16b, respectively, which provide smaller contributions. The emergence of this constraint implies that the one established within the 2nd mode simplifies to the equilibration of the two directions of the 14th reaction. The third fastest mode along the Lactate path is the 6th and in the context of the ANLS case denotes the equilibration among the 16th, 15th and 7th reactions:

$$\begin{array}{c} z_{16}^{6}\left(R^{16f}-R^{16b}\right)+z_{15}^{6}\left(R^{15f}-R^{15b}\right)-z_{7}^{6}R^{7}\approx 0 \end{array}$$ (14)

while in the context of the NALS case denotes the equilibration among the 16th, 15th and 9th reactions:

$$\begin{array}{c} z_{16}^{6}\left(R^{16f}-R^{16b}\right)+z_{15}^{6}\left(R^{15f}-R^{15b}\right)-z_{9}^{6}R^{9}\approx 0 \end{array}$$ (15)

This mode refers to *Lac*^*a*^ and *Lac*^*n*^. As a result, the *Lac*^*n*^-consuming reaction 16b and the *Lac*^*a*^-consuming reaction 15f provide the largest contributions to *τ*_6_, while their reverse rates tend to diminish their influence; i.e., they tend to destroy this constraint. The slowest of the fast modes along the Lactate path is the 7th, which in both the ANLS and NALS cases denotes the equilibration among the 11th, 12th and 16th reactions:

$$\begin{array}{c} -{\hat{z}}_{16}^{4}\left(R^{16f}-R^{16b}\right)+{\hat{z}}_{12}^{4}\left(R^{12f}-R^{12b}\right)+{\hat{z}}_{11}^{4}\left(R^{11f}-R^{11b}\right)\approx 0 \end{array}$$ (16)

in which the net rate of reaction 16 provides a higher order correction. This mode refers mainly to *Lac*^*e*^, as indicated by the *Po*, which is reactant of reactions 11b and 12f that provide the largest contributions to *τ*_7_.

A schematic representation of these constraints that develop along the Lactate path is shown in Fig 3. As with the *Glc*-related constraints, the four *Lac*-related constraints involve all five compartments along the Lactate path and all means of transport and diffusion among them, with the contribution of the 13th reaction being negligible, since its influence is overshadowed by that of the 14th and 16th reactions; see S1 Text.

The system evolves on a surface of equilibria, approximated by these constraints, on which the slow dynamics become the characteristic ones. Having established, from the data displayed in Tables 1 and 2, that the species related the most to the slow dynamics are *Glc*^*a*^, *Glc*^*n*^, *Lac*^*a*^ and *Lac*^*n*^ (since they relate the least to the fast dynamics), we proceed forward by analyzing the metabolic profiles of neurons and astrocytes.

### Slow dynamics

Interested in examining the dynamics of neurons and astrocytes interaction, the slow evolution of the system during neuronal activation is analyzed with the CSP tools. The capability to algorithmically determine the underlying physics of the slowly evolving system, within the constraints imposed by the exhausted modes, is demonstrated in this section.

#### Characteristic dynamics and driving reactions during the slow evolution

As stated in the previous section, during neuronal activation (periods *P*_1_ and *P*_2_), only the 9th and 10th modes are active in the ANLS case and only the 8th, 9th and 10th modes are active in the NALS case. Given that *τ*_9_ < *τ*_10_ in the ANLS case, the slow evolution of the system is characterized by *τ*_9_. However, since *τ*_8_ ≈ *τ*_9_ < *τ*_10_ in the NALS case, the slow evolution of the system is characterized by both *τ*_8_ and *τ*_9_. Tables 3 and 4 display CSP diagnostics (*Po*, *TPI*) of the slow modes in the ANLS and NALS cases, respectively, computed at *t* = 1100 *s*.

**Table 3:** CSP diagnostic tools for the 2 active modes in the ANLS case.

Mode	λ [1/s]	*τ* [s]	*Po*		*TPI*	
9	-6.197E-03	1.614E+02	*Lac*^*n*^	0.472	18	-33.5%
			*Lac*^*a*^	0.314	17	-19.0%
			*Lac*^*int*^	0.192	16f	-17.9%
					16b	17.7%
10	-1.880E-03	5.318E+02	Glc^n^	0.526	4b	-17.1%
			Glc^a^	0.280	2b	-16.8%
			Glc^int^	0.177	4f	15.4%
					6b	-11.7%
					6f	11.2%
					1b	-8.5%
					2f	8.4%
					5f	-3.2%

The largest *Po* and *TPI* for the slow 9th and 10th modes during neuronal activation for the ANLS case; *t* = 1100 *s*.

**Table 4:** CSP diagnostic tools for the 3 active modes in the NALS case.

Mode	λ [1/s]	*τ* [s]	*Po*		*TPI*	
8	-6.855E-03	1.458E+02	Glc^*n*^	0.564	5f	-27.3%
			Glc^*a*^	0.337	6b	-18.5%
			Glc^*int*^	0.090	6f	15.6%
					5b	-11.2%
					4b	-6.5%
					4f	5.7%
					2b	-5.3%
9	-5.694E-03	1.756E+02	Lac^*n*^	0.508	18	-35.3%
			Lac^*a*^	0.286	17	-21.4%
			Lac^*int*^	0.185	16f	-12.0%
					16b	11.9%
10	-1.733E-03	5.770E+02	Glc^*a*^	0.435	4b	-17.0%
			Glc^*n*^	0.406	2b	-15.9%
			Glc^*int*^	0.145	4f	15.2%
					6b	-9.2%
					6f	8.9%
					5f	-8.3%
					1b	-8.1%
					2f	8.0%

The largest *Po* and *TPI* for the slow 8th, 9th and 10th modes during neuronal activation for the NALS case; *t* = 1.100 *s*.

It is shown in Table 3 that in the ANLS case the pointed variables and the reactions contributing to the related *τ*_*i*_ of the two slow modes refer to either the Lactate path (9th mode) or to the Glucose path (10th mode). As it will be shown next, this feature is due to the fact that these two modes are practically decoupled. Naturaly, the pointed variables in the two modes are the ones related the least with the fast dynamics, as it is evident by the results displayed in Table 1.

Regarding the 9th mode, the *Lac*^*n*^-consuming reaction 18 and the *Lac*^*a*^-consuming reaction 17, which represent the *Lac* oxidation in neurons and astrocytes, respectively, account for more than 50% of the total contribution to the generation of *τ*_9_. On the basis of their negative TPI, it is concluded that these reactions tend to drive the system to a fixed point. On the other hand, the contributions to *τ*_9_ of the two directions of the 16th reaction, which represent the transport of *Lac* from interstitium to neuron and vice versa, are of opposing nature; the *Lac*^*int*^-consuming forward direction promoting the movement towards a fixed point and the backward opposing it. Their contributions cancel each other, so that the net influence of the 16th reaction is negligible. The dominance of the 17th and 18th reactions in the generation of *τ*_9_ is reflected in the large *Po* value of their reactants *Lac*^*a*^ and *Lac*^*int*^. This dominance implies that the duration of the neuronal activation is mainly controlled by these two reactions. For example, a decrease of the *Lac* oxidation rate via reaction 18 will lead to the increase of *τ*_9_; i.e., it will lead to a slower evolution of the process. This influence of the 18th reaction will be demonstrated later by properly perturbing *R*^18^.

The results displayed in Table 3 also show that in the context of the 10th mode in the ANLS case, significant contributions to the generation of *τ*_10_ are provided by the *Glc*^*int*^-consuming reaction 4*b*, the *Glc*^*n*^-consuming reaction 6*b* and the *Glc*^*a*^-consuming reaction 5*f*. Other reactions provide additional contributions, mainly via the already established constraints. Counting the net contribution of each reaction (by adding the contributions of the forward and backward direction), the most significant influence originates from the 1st reaction, followed by the 2nd and then by the 4th.

Table 4 shows that, as in the ANLS case, the pointed variables and the reactions contributing to the related *τ*_*i*_ of the three slow modes in the NALS case refer to either the Lactate path (9th mode) or to the Glucose path (8th and 10th mode). The dynamics of the two slowest modes (9th and 10th) are similar to the equivalent modes in the ANLS case with small variations. However, in contrast to the ANLS case, in the NALS case the 8th mode is accounted in the slow component of the vector field and its dynamics characterise the evolution of the system, along with the dynamics of the 9th mode (since *τ*_8_ ≈ *τ*_9_). It is shown in Table 4 that the largest net contribution in generating *τ*_8_ originates from the *Glc*^*a*^-consuming 5th reaction, while the net contributions of the 6th and 4th reactions are much smaller. In comparison to the ANLS case, the dynamics of the slowest 10th mode in the NALS case mainly differs in that *Glc*^*a*^ is more pointed and *Glc*^*n*^ is less, while the differences in the 9th mode are minor.

Comparing the slow dynamics of the ANLS and NALS cases, Tables 3 and 4 show that the slow dynamics along the *Glc*-path shifts from neurons towards astrocytes, as it is evident by the sum of the *CSP Pointer* of *Glc*^*n*^ and *Glc*^*a*^ due to the slow modes ($D_{Glc^{n}}^{9+10}$ = 0.526 and $D_{Glc^{a}}^{9+10}=0.280$ in the ANLS case and $D_{Glc^{n}}^{8+9+10}$ = 0.970 and $D_{Glc^{a}}^{8+9+10}=0.772$ in the NALS case; i.e., a 84.5% and a 175.7% increase of the sum of slow Pointers of *Glc*^*n*^ and *Glc*^*a*^, respectively). This shift of the slow dynamics along the *Glc*-path is accompanied by an opposite shift of the fast dynamics; i.e. from astrocytes towards neurons. This feature is clearly evident in Fig 3, by the significantly reduced contribution of the *Glc*^*a*^-related 5th reaction in the established constraints in the NALS case and the ensuing reduced influence of *Glc*^*a*^ in the fast dynamics. In turn, as shown in Tables 1 and 2, this last feature results in a reduced involvement in the fast dynamics of the *Glc*^*a*^-consuming 7th reaction, that couples the *Glc*-path to the *Lac*-path.

The decoupling of the *Po* and *TPI* diagnostics along the *Glc*- and *Lac*-paths in the context of the slow modes in the ANLS and NALS cases, as indicated by Tables 3 and 4, is reasonable for the *Glc*-path, since the concentration of *Glc* in the various compartments is not dependent on that of *Lac*, as shown in Fig 1. However, this is not that obvious for the *Lac*-path, since it is influenced by the *Glc*-path via the 7th and 9th reactions. In order to investigate this issue further, the reactions contributing the most to the evolution of *Glc*^*a*^, *Glc*^*n*^, *Lac*^*a*^ and *Lac*^*n*^ (thereafter, *Glc*^*a*,*n*^ and *Lac*^*a*,*n*^) will be identified, with the use of the Importance Index *II*. The results displayed in Table 5 identify (i) the reactions that influence most the evolution of *Glc*^*a*,*n*^ and *Lac*^*a*,*n*^ and (ii) the corresponding values of *II*. The positive (negative) sign of the contribution of a reaction displayed in Table 5, denotes its effect in producing (consuming) the related species.

**Table 5:** Contributions of the reactions controlling the slow evolution of *Glc*^*a*^, *Glc*^*n*^, *Lac*^*a*^ and *Lac*^*n*^ in the ANLS and NALS case.

	Species	Slow system (*II* identifications in decreasing order)
**ANLS**	*Glc*^*a*,*n*^	$\begin{array}{ll} \frac{d[\cdot ]}{dt} & \approx f\left(+{\text{R}}^{1f},-{\text{R}}^{1b},+{\text{R}}^{2f},-{\text{R}}^{7},-R^{2b},+R^{4f},-R^{4b},-R^{9}\right)\\ & \begin{array}{ll} & +0.209-0.151+0.149-0.107-0.092+0.069-0.057-0.029 \end{array} \end{array}$
	*Lac*^*a*,*n*^	$\begin{array}{ll} \frac{d[\cdot ]}{dt} & \approx f\left(+{\text{R}}^{7},-{\text{R}}^{18},-{\text{R}}^{17},-R^{16f},+R^{16b},+R^{9}\right)\\ & \begin{array}{ll} & +0.295-0.207+0.143-0.081-0.078+0.078 \end{array} \end{array}$
**NALS**	*Glc*^*a*^	$\begin{array}{ll} \frac{d[\cdot ]}{dt} & \approx f\left(+{\text{R}}^{5b},-{\text{R}}^{6f},+{\text{R}}^{6b},-{\text{R}}^{7},-{\text{R}}^{5f},+R^{1f},+R^{2f}\right)\\ & \begin{array}{ll} & +0.225-0.201+0.182-0.126-0.125+0.027+0.027 \end{array} \end{array}$
	*Glc*^*n*^	$\begin{array}{ll} \frac{d[\cdot ]}{dt} & \approx f\left(+{\text{R}}^{1f},-{\text{R}}^{1b},+{\text{R}}^{2f},+R^{6f},-R^{9},-R^{6b},-R^{2b},+R^{4f},-R^{4b}\right)\\ & \begin{array}{ll} & +0.175-0.130+0.127+0.098-0.092-0.089-0.083+0.067-0.056 \end{array} \end{array}$
	*Lac*^*a*,*n*^	$\begin{array}{ll} \frac{d[\cdot ]}{dt} & \approx f\left(+{\text{R}}^{9},-{\text{R}}^{18},-{\text{R}}^{17},+R^{7},-R^{16f},+R^{16b}\right)\\ & \begin{array}{ll} & +0.313-0.236-0.133+0.089-0.054+0.055 \end{array} \end{array}$

Identification of the reactions contributing the most to the slow evolution of the *Glc*^*a*^, *Glc*^*n*^, *Lac*^*a*^ and *Lac*^*n*^ by *II*, during the neuronal activation for the ANLS (top) and the NALS (bottom) cases; *t* = 1100*s*. Reactions in bold denote contributions larger than 10%. Only the largest contributions are displayed. Numbers denote the corresponding *II* value.

Table 5 shows that in the ANLS case the evolution of *Glc*^*a*,*n*^ is mainly determined by the *R*^1*f*^ (*Glc* transport from serum to endothelium), *R*^1*b*^ (*Glc* transport from endothelium to serum) and *R*^2*f*^ (*Glc* transport from endothelium to basal lamina). Smaller influences of the rates *R*^7^ and *R*^2*b*^ towards depleting *Glc*^*a*,*n*^ are also reported, while the even smaller influences of the two rates *R*^4*f*^ and *R*^4*b*^ cancel each other. Regarding *Lac*^*a*,*n*^, Table 5 shows that its evolution is mainly determined by the rates *R*^7^ (glycolytic rate in astrocytes) and *R*^18^ and *R*^17^ (*Lac* oxidative rate in neurons and astrocytes, respectively); the first tending to increase *Lac*^*a*,*n*^, while the latter two to decrease it. Smaller influences are reported by *R*^6*f*^ and *R*^6*b*^, which cancel each other. Table 5 shows that *R*^9^ has a small influence in driving both *Glc*^*a*,*n*^ and *Lac*^*a*,*n*^.

Regarding the reactions that drive *Glc*^*a*,*n*^ and *Lac*^*a*,*n*^ in the NALS case, the differences in the results displayed in Table 5 relative to the ANLS case are mainly due to the decreased influence of fast dynamics on *Glc*^*a*^. As it is shown in Table 5, the evolution of *Glc*^*a*^ is now mainly determined by reactions that either consume it or produce it (*R*^5*f*^, *R*^5*b*^, *R*^7^), while the recorded net contributions by the 1st, 2nd and 6th reactions are smaller. The reactions that mainly determine the evolution of *Glc*^*n*^ in the NALS case are similar to those identified in the ANLS case. The main difference is the decreased influence of the 7th reaction and the increased one of the 9th. This is also the main difference in the reactions that drive *Lac*^*a*,*n*^; apart from *R*^18^ and *R*^17^ that play a major role in the ANLS case, now *R*^9^ exhibits the strongest influence, while that of *R*^7^ is significantly diminished.

#### From the ANLS to the NALS configuration

**Fig 4:**
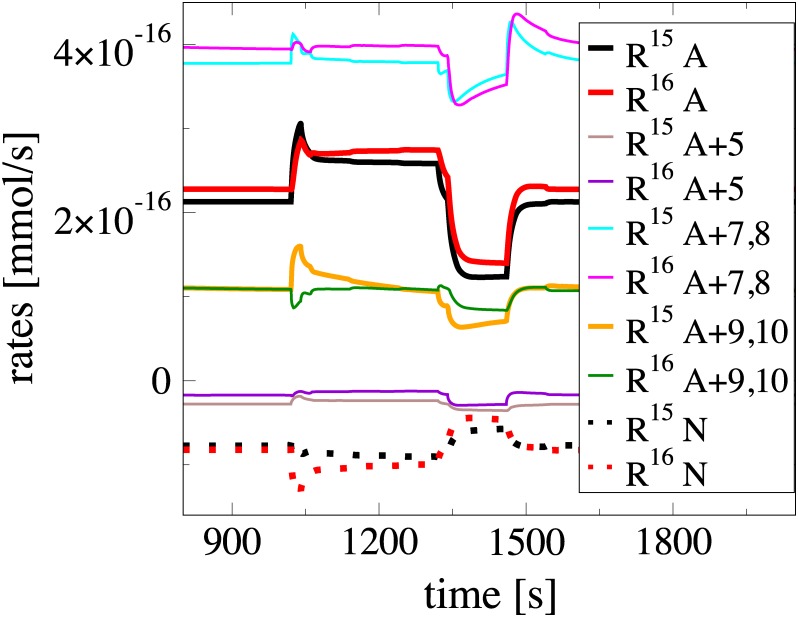
Evolution of *R*^15^ and *R*^16^ rates when applying the modifications leading from ANLS to NALS case, separately. Evolution of the net rates *R*^15^ = *R*^15,*f*^ − *R*^15,*b*^ and *R*^16^ = *R*^16,*f*^ − *R*^16,*b*^, related to the *Lac* flow from astrocytes to interstitium and from there to neurons, in the ANLS (A) and NALS (N) cases. In the ANLS case *R*^15^ and *R*^16^ are positive, while in the NALS case are negative. The net rates *R*^15^ and *R*^16^ are also displayed in the case were each of the modifications leading from the ANLS to the NALS configuration is implemented separately. These modifications relate to changes in the parameters of reactions 3, 5, 7, 8, 9 and 10; the profiles related to the modification of reaction 3 are not shown, since the differences with the ANLS case are negligible.

The differences between the ANLS and NALS configurations lies in the different values of the parameters of reactions 3, 5, 7, 8, 9, 10, 17 and 18. In order to investigate the influence of these different values, each of the changes in the value of the parameters, leading from the ANLS to the NALS configuration, was implemented separately. It was shown that only the changes in the parameters of the reactions 5, 7, 8, 9 and 10 produce significant deviations from the solution obtained with the ANLS model. The influence of these changes is demonstrated in Fig 4, where the evolution of the net rates *R*^15^ = *R*^15,*f*^ − *R*^15,*b*^ and *R*^16^ = *R*^16,*f*^ − *R*^16,*b*^ is displayed. These two net rates relate to the *Lac* flow from astrocytes to interstitium and from there to neurons, in the ANLS (A) and NALS (N) cases. As shown in the figure, in the ANLS case *R*^15^ and *R*^16^ are positive, while in the NALS case are negative. In addition, Fig 4 displays the evolution of these two rates in the case where each of the changes in the parameters of the reactions that lead from the ANLS to the NALS configuration is implemented separately; e.g., “*R*^15^ *A* + 7, 8” indicates the profile of *R*^15^ when the ANLS configuration is considered with the exception of the parameter in the rate of reactions 7 and 8 for which the change that leads to the NALS configuration is accounted for (see S1 Text for a list of all changes in the parameters that lead from the ANLS to the NALS configuration).

It is demonstrated in Fig 4 that the most influential change in the shift from the ANLS to the NALS configuration is produced by the parameter that modifies *R*^5^, followed by the one modifying *R*^9^ and *R*^10^. In contrast, the change in the parameter that modifies *R*^7^ and *R*^8^ shifts the ANLS configuration away from the NALS one. This finding is in agreement with the substantially different influence of *R*^5^ in the fast dynamics of the ANLS and NALS cases, discussed previously; i.e., in the ANLS case *R*^5^ is fast enough that allows the coupling of the *Glc* and *Lac* pathways via the 7th reaction (so that the flow of *Lac* is directed from astrocytes to neurons), while in the NALS case *R*^5^ is slow enough, so that the coupling between the two pathways is mainly established via the 9th reaction (so that the flow of *Lac* is directed from neurons to astrocytes).

### Assessment of CSP diagnostics

**Fig 5:**
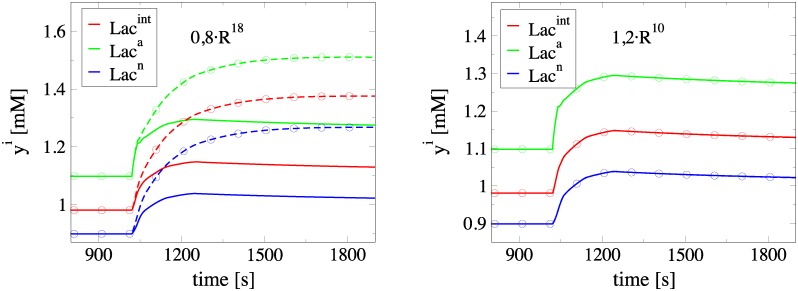
Response of *Lac*^*int*,*a*,*n*^ in decrease/increase of *R*^18^/*R*^10^. Evolution of *Lac*^*int*,*a*,*n*^ during neuronal activation. Comparison of the solution under normal conditions (solid lines) with the one under perturbed rates conditions (dashed lines with circles). Left: Decrease of *R*^18^ by 20%. Right: Increase of *R*^10^ by 20%.

The conclusions reached on the basis of the various CSP diagnostic tools will be assessed here, by perturbing the rates of selected reaction rates. For simplicity, only the ANLS case will be considered; similar conclusions are reached in the NALS case. In particular, consider the rate *R*^18^, which does not contribute to the generation of the eight constraints, as shown in Table 1. In addition, the *TPI* results in Table 3 suggested that the rate *R*^18^ has a significant contribution to the generation of the characteristic time scale *τ*_9_. Finally, the *II* results in Table 5 suggested that *R*^18^ tends to decrease *Lac*^*a*,*n*^. Therefore, a decrease of the rate constant of *R*^18^ is expected to increase *τ*_9_ and *Lac*^*a*,*n*^. Indeed, as it is shown in the left part of Fig 5, a 20% decrease of *R*^18^ causes *Lac*^*n*^, *Lac*^*a*^ and *Lac*^*int*^ to reach a constant value at *t* ≈ 1805 *s*, while in the unperturbed case this state is achieved faster, at *t* ≈ 1235 *s*. In addition, the levels of *Lac*^*a*,*n*^ are higher in the perturbed case, as expected. On the other hand, the increase of the rate *R*^10^ by 20% has negligible influence on the evolution of *Lac*^*a*,*n*^, as shown at the right part of Fig 5. This is an expected result, since *R*^10^ has a negligible contribution to the eight constraints, to *τ*_9_ and to the slow evolution of the system; see Tables 1, 3 and 5.

A further demonstration of the usefulness of the CSP diagnostic tools emerges from the physical interpretation of the *II* results displayed in Table 5. In particular, consider the response of *Lac*^*a*,*n*^ and *Glc*^*a*,*n*^ when subjected to perturbations of *R*^18^, *R*^9^, *R*^1*f*^ and *R*^3*f*^ during neuronal activation. Such perturbations might affect the *M* constraints or the reduced model; see Eq (3). According to the *API* values in Table 1, *R*^1*f*^ is the only rate from this set that influences the constraints, via the 4th and the 5th modes that relate to *Glc*^*e*^ and *Glc*^*int*^. According to the *II* values in Table 5 that assesses the influence of the reactions in the sow model, (i) *R*^18^ has the largest contribution towards decreasing *Lac*^*a*,*n*^ levels, while has negligible influence in the *Glc*^*a*,*n*^, (ii) *R*^9^ exhibits a small influence in increasing *Lac*^*a*,*n*^ levels and a smaller influence in decreasing *Glc*^*a*,*n*^ levels, (iii) *R*^1*f*^ is the major contributor towards increasing *Glc*^*a*,*n*^, but has negligible influence in the evolution of *Lac*^*a*,*n*^ and (iv) *R*^3*f*^ has a negligible contribution to the evolution of all variables.

**Fig 6:**
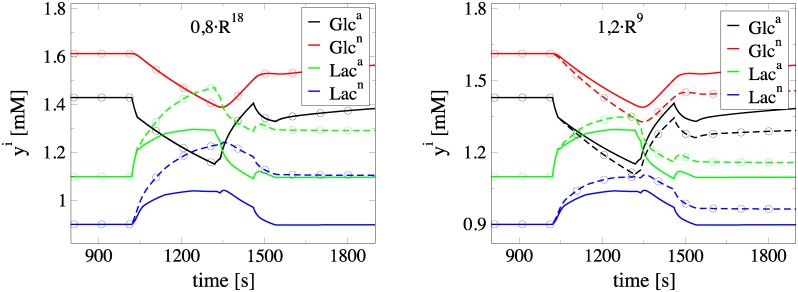
Response of *Glc*^*a*,*n*^ and *Lac*^*a*,*n*^ in decrease/increase of *R*^18^/*R*^9^. Evolution of *Glc*^*a*,*n*^ and *Lac*^*a*,*n*^ during neuronal activation. Comparison of the solution under normal conditions (solid lines) with the one under perturbed rates conditions (dashed lines with circles). Left: Decrease of *R*^18^ by 20%. Right: Increase of *R*^9^ by 20%.

**Fig 7:**
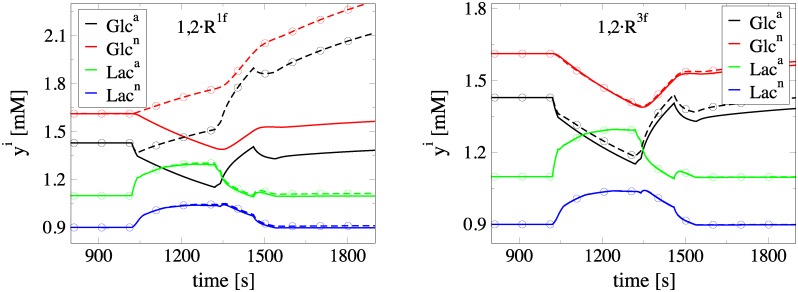
Response of *Glc*^*a*,*n*^ and *Lac*^*a*,*n*^ in increase of *R*^1*f*^ and *R*^3*f*^. Evolution of *Glc*^*a*,*n*^ and *Lac*^*a*,*n*^ during neuronal activation. Comparison of the solution under normal conditions (solid lines) with the one under perturbed rates conditions (dashed lines with circles). Left: Increase of *R*^1*f*^ by 20%, Right: Increase of *R*^3*f*^ by 20%.

The predicted influence of perturbations of *R*^18^, *R*^9^, *R*^1*f*^ and *R*^3*f*^ is demonstrated in Figs 6 and 7. Specifically, in the left part of Fig 6 it is shown that the decrease of *R*^18^ leads to increased *Lac*^*a*,*n*^ levels, but leaves *Glc*^*a*,*n*^ unaffected. On the right part of the same figure, it is shown that the increase of *R*^9^ leads to increased *Lac*^*a*,*n*^ levels and decreased *Glc*^*a*,*n*^ levels. Note that the response of *Lac*^*a*,*n*^ to a perturbation of *R*^18^ is more intense than that of *Glc*^*a*,*n*^ and *Lac*^*a*,*n*^ to a similar perturbation of *R*^9^, in accordance to the related *II* results displayed in Table 5. Also, in the left part of Fig 7 it is shown that the decrease of *R*^1*f*^ leads to increased *Glc*^*a*,*n*^ levels, but leaves *Lac*^*a*,*n*^ levels unaffected. Finally, in the right part of the same figure it is shown that an increase of *R*^3*f*^ has a negligible influence to both *Glc*^*a*,*n*^ and Lac^*a*,*n*^, as predicted.

### Investigating an exercise scenario

It was shown previously that a decisive factor on whether the ANLS or NALS configuration will develop is whether the 5th reaction contributes to the fast dynamics or not. In particular, it was shown that in the context of the ANLS (NALS) configuration the 5th reaction contributes (does not contribute) to the fast dynamics, allowing thus the major coupling between the *Glc* and the *Lac* paths to be established in astrocytes via the 7th reaction (in neurons via the 9th reaction). As a result, the *Lac* flow is directed from astrocytes to neurons in the ANLS case and in the opposite direction in the NALS case. These conclusions were reached for normal conditions (NC); among others, low *Lac* concentration in serum (*Lac*^*s*^ = 1 *mM*).

However, under exercise conditions (EC) the *Lac* concentration in the serum reaches large values, about 15 − 25 *mM* (“all-out” maximal exertion) [123, 124]. A high *Lac* concentration in serum will certainly influence the concentrations along the *Lac*-path. Nonetheless, this factor will leave the concentrations of *Glc* unaffected, since the *Glc*-path is not coupled to the *Lac*-path. As a result, it is not expected to influence the degree to which the 5th reaction contributes to the fast dynamics; i.e., the conditions under which the *Glc*-path dynamics favor the development of ANLS or NALS. The only feature that might be affected is the flow direction of *Lac* between astrocytes and neurons, given the changes in the concentrations along the *Lac*-path that result by the high *Lac* concentration in the serum. These issues will be examined next.

**Fig 8:**
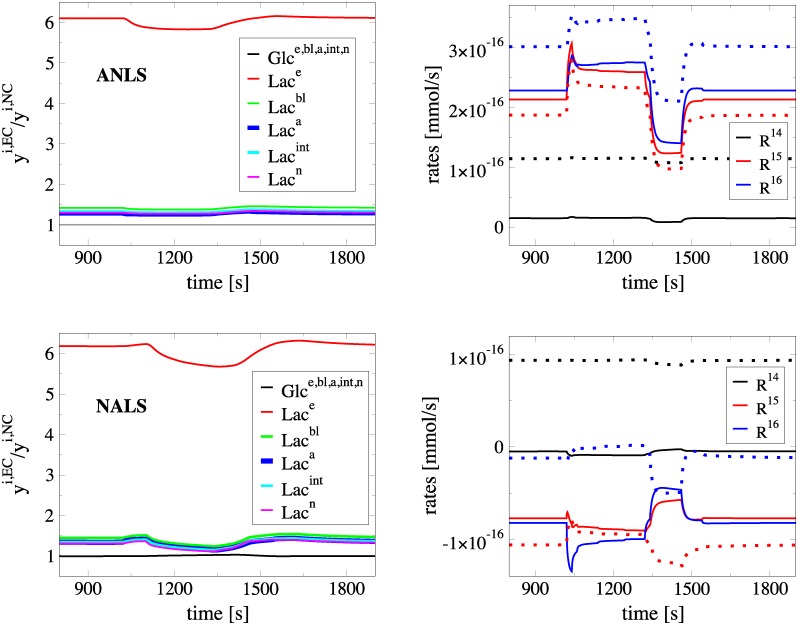
Comparison of the metabolites concentrations and the rates *R*^14^, *R*^15^ and *R*^16^ in moderate exercise conditions with the normal ones. Ratio *y*^*i*,*EC*^/*y*^*i*,*NC*^ of various concentrations under exercise (*y*^*i*,*EC*^) and normal (*y*^*i*,*NC*^) conditions (left) and profiles of the net reaction rates *R*^14^ = *R*^14,*f*^ − *R*^14,*b*^, *R*^15^ = *R*^15,*f*^ − *R*^15,*b*^ and *R*^16^ = *R*^16,*f*^ − *R*^16,*b*^ (right), during neuronal activation for the ANLS (top) and NALS (bottom) cases, for the moderate exercise scenario. In the figures on the right panels solid/dashed lines denote NC/EC, respectively.

In the exercise scenario considered here, the serum *Lac* concentration was increased to *Lac* = 20 *mM* (from *Lac* = 1 *mM* in NC). As expected, high serum *Lac* conditions, introduce significant changes to all *Lac*^*i*^, but leave unaffected those of all *Glc*^*i*^ in both ANLS and NALS cases. This is displayed in the left panels of Fig 8, where the ratio of the concentrations computed under exercise conditions (EC) and under normal conditions (NC), *y*^*i*,*EC*^/*y*^*i*,*NC*^, is displayed. This ratio equals unity for *Glc*^*i*^ in all the 5 compartments, while it is slightly larger than unity for the *Lac*^*i*^ in all compartments, with the exception of the ratio for *Lac*^*e*^ which is around 6.

**Fig 9:**
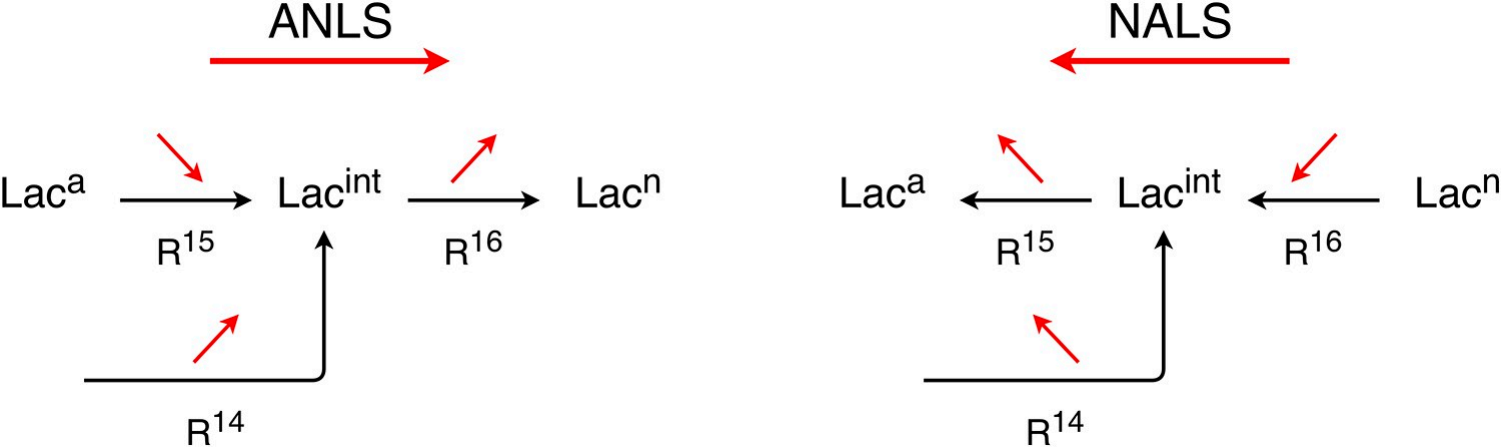
Schematic presentation of the changes in the rates *R*^14^, *R*^15^ and *R*^16^ in moderate exercise conditions compared with the normal ones. A schematic presentation of the changes in the net rates *R*^14^, *R*^15^ and *R*^16^ caused by the increased *Lac* concentration in serum in the ANLS (left) and NALS (right) cases at the moderate exercise scenario; in comparison to the NC case, *R*^14^ increases in both ANLS and NALS, *R*^15^ decreases in the ANLS case and increases in the NALS case, while *R*^16^ increases in the ANLS case and decreases in the NALS case.

The increased concentrations of *Lac*^*i*^ modify the flow of lactate between astrocytes and neurons. As shown in the right panels of Fig 8, the high serum *Lac* concentration causes the rate of *Lac*-transport from astrocytes to interstitium (*R*^15^ = *R*^15,*f*^ − *R*^15,*b*^ > 0) to decrease and from interstitium to neurons (*R*^16^ = *R*^16,*f*^ − *R*^16,*b*^ > 0) to increase in the ANLS case. In contrast, in the NALS case the *Lac*-transport from neurons to interstitium (-*R*^16^ > 0) decreases and from interstitium to astrocytes (-*R*^15^ > 0) increases. Fig 8 shows that in both the ANLS and NALS cases the flow of lactate from basal lamina to intertitium (*R*^14^) increases. While in the ANLS case *Lac* flows from basal lamina to intertitium (*R*^14^ > 0) in both NC and EC, in the case of NALS the minor flow from intertitium to basal lamina (*R*^14^ < 0) in NC is significantly reversed in EC. A schematic presentation of the changes in the net rates *R*^14^, *R*^15^ and *R*^16^ is displayed in Fig 9. Clearly, the increased values of *R*^16^ in the ANLS case and of −*R*^15^ in the NALS case are in agreement with the nature of ANLS and NALS assumptions, respectively; since *Lac* is transported towards the neurons in the ANLS case and towards the astrocytes in the NALS case increases. However, the decreased values of *R*^15^ in the ANLS case and of −*R*^16^ in the NALS case are in disagreement; since the transport of *Lac* from astrocytes in the ANLS case and that from neurons in the NALS case decreases. In fact, as shown by the profile of *R*^16^ in the NALS case, at about *t* = 1300 *s*, *Lac* is transported to neurons (*R*^16^ > 0) and not from them, as the NALS hypothesis dictates.

In summary, it was shown that an increased serum *Lac* concentration resulted in an increased transport of *Lac* to neurons in the ANLS case, mainly due to the increased diffusion from basal lamina, accompanied by a decreased transport out of astrocytes. In contrast, an increased transport of *Lac* to astrocytes was recorded in the ANLS case, also due to the increased diffusion from basal lamina, accompanied by a decreased transport out of neurons. It can be shown that these features are in full agreement with the equilibrations that develop in the context of the 2nd and 3rd modes, as they are stated in Tables 1 and 2, which mainly involve the rates *R*^16^, *R*^15^ and *R*^14^ and to a lesser degree *R*^13^ in normal and exercise conditions and the rate *R*^12^ only in exercise conditions.

## Discussion

Fast and slow dynamics influence the response of the system in both the ANLS and NALS frameworks in two distinct manners. The fast dynamics are responsible for the constraints that are established in phase space, within which the system evolves driven by processes responsible for the slow dynamics.

Constraints develop along both the Glucose and Lactate paths. Only one of these constraints couples the two paths; i.e., the Lactate to the Glucose path, while the latter is uncoupled to the former, see Fig 1. Along the Glucose path in both the ANLS and NALS cases the following constraints related to *Glc*-transport develop:

1. from endothelium to basal lamina (*R*^2*f*^ − *R*^2*b*^) and from there to interstitium (*R*^4*f*^ − *R*^4*b*^),
2. from serum to endothelium (*R*^1*f*^ − *R*^1*b*^) and from there to basal lamina (*R*^2*f*^ − *R*^2*b*^), with the contribution of a higher order correction from the *Glc*-transport in and out of the interstitium (*R*^4*f*^ − *R*^4*b*^, *R*^5*f*^ − *R*^5*b*^, *R*^6*f*^ − *R*^6*b*^); the latter contribution based on the previous establishment of the constraint in (i),
3. from interstitium to neurons (*R*^6*f*^ − *R*^6*b*^), from serum to endothelium (*R*^1*f*^ − *R*^1*b*^) and only in the NALS case from astrocyte to interstitium (*R*^5*f*^ − *R*^5*b*^), with the contribution of a higher order correction from the *Glc*-transport from basal lamina to interstitium (*R*^4*f*^ − *R*^4*b*^) due to the constraints established previously in (i) and (ii), and
4. only in the ANLS case, from astrocytes to interstitium (*R*^5*f*^ − *R*^5*b*^) and from there to neurons (*R*^6*f*^ − *R*^6*b*^), with the glycolytic rate in astrocytes *R*^7^ contributing as a higher order correction.

As Fig 3 shows, these constraints involve all five compartments along the Glucose path. Along the Lactate path in both the ANLS and NALS cases the following constraints related to *Lac*-transport develop:

1. from basal lamina to interstitium (*R*^14*f*^ − *R*^14*b*^), with the *Lac*-transport from interstitium via reactions 15 (*R*^15*f*^ − *R*^15*b*^) and 16 (*R*^16*f*^ − *R*^16*b*^) providing a higher order correction,
2. from astrocytes to interstitium (*R*^15*f*^ − *R*^15*b*^) and from there to neurons (*R*^16*f*^ − *R*^16*b*^),
3. from astrocytes to interstitium (*R*^15*f*^ − *R*^15*b*^) and from there neurons (*R*^16*f*^ − *R*^16*b*^), with a small contribution from reactions 7 and 9 from the Glucose path in the ANLS and NALS cases, respectively, and
4. from serum to endothelium (*R*^11*f*^ − *R*^11*b*^) and from there to basal lamina (*R*^12*f*^ − *R*^12*b*^), with only a small contribution from reaction 16 which is possible after the equilibria in (i), (ii) and (iii) are established.

As with the constraints related to the Glucose path, Fig 3 shows that these constraints involve all five compartments along the Lactate path.

Clearly, the major difference in the ANLS and NALS cases is the contribution to the fast dynamics of *Glc*-transport from astrocytes to interstitium (*R*^5*f*^ − *R*^5*b*^). In the ANLS case this *Glc*-transport participates in both the fast dynamics and in the ensuing related constraints (large TPI and API in the 4th, 5th and 8th modes; see Table 1). However, in the NALS case this participation is greatly diminished (small TPI and API in the 4th and 5th modes, while the 8th mode is now a slow one; see Table 2). These features suggest that the strong coupling ot the *Lac*- to the *Glc*-path that is established in the ANLS case (mainly via the 7th reaction inside the asrtocytes, in the context of the 6th and 8th modes) diminishes significantly in the NALS case (where this coupling is now established mainly via the 9th reaction inside the neurons, in the context of only the 6th mode).

The finding that—due to the behavior of *R*^5^—the *Lac*- to the *Glc*-path coupling is mainly established inside the astrocytes in the ANLS case and inside the neurons in the NALS case determines the conditions promoting the ANLS or NALS development. In the ANLS case *R*^5^ participates in the equilibria of the 4th and 5th modes, which allow the *Glc* concentrations in both the astrocytes and neurons to be coupled directly with that in the serum. The formation of *Lac* from *Glc* is more intense in astrocytes (*R*^7^) than in neurons (*R*^9^), so that the typical for the ANLS case flow of *Lac* from astrocytes to neurons is generated. In the NALS case the participation of *R*^5^ to the equilibria of the 4th and 5th modes is significantly reduced, so that only the *Glc* concentration in neurons is coupled directly with that in the serum. Therefore, the formation of *Lac* from *Glc* in neurons (*R*^9^) dominates, so that the typical for the NALS case flow of *Lac* from neurons to astrocytes is generated.

The influence of the fast dynamics in determining the development of ANLS or NALS is fully reflected in the slow dynamics that characterize the evolution of the system within the established constraints. In particular, as shown in Table 5, in the ANLS case the *Glc*^*a*,*n*^ level is mainly determined by the rate of reactions in the first half of the *Glc*-path (*R*^1^, *R*^2^) and the glycolytic rates in astrocytes (*R*^7^), while the *Lac*^*a*,*n*^ level is mainly determined by rates in the second half of the *Lac*-path (*R*^17^ and *R*^18^) and by the rate *R*^7^. In essence, the glycolytic rate in astrocytes (*R*^7^) couples the *Lac* oxidative rates in astrocytes and neurons (*R*^17^ and *R*^18^) with the *Lac* inflow rate from the serum, via reactions at the start of the *Glc*-path (*R*^1^, *R*^2^). As a result, the flow along the *Lac* pathway in the ANLS case is directed from astrocytes to interstitium (*R*^15^ = *R*^15,*f*^ − *R*^15,*b*^ > 0) and from there to neurons (*R*^16^ = *R*^16,*f*^ − *R*^16,*b*^ > 0). In contrast, Table 5 shows that in the NALS case the same coupling is now established mainly via the glycolytic rate in neurons (*R*^9^), while the glycolytic rate in astrocytes (*R*^7^) exhibits a smaller influence. In this case the flow along the *Lac* pathway is directed from neurons to interstitium (*R*^16^ = *R*^16,*f*^ − *R*^16,*b*^ < 0) and from there to astrocytes (*R*^15^ = *R*^15,*f*^ − *R*^15,*b*^ < 0). In both cases, the direct coupling of the *Lac* oxidative rates in astrocytes and neurons (*R*^17^ and *R*^18^) with the *Lac* inflow rate from the serum via the reactions in the first of the *Lac*-path (*R*^12^ and *R*^13^) has no significant influence. Finally, in agreement to the model depicted schematically in Fig 1, Table 5 shows that in both the ANLS and NALS cases *Glc*^*a*,*n*^ is independent from *Lac*^*a*,*n*^.

**Fig 10:**
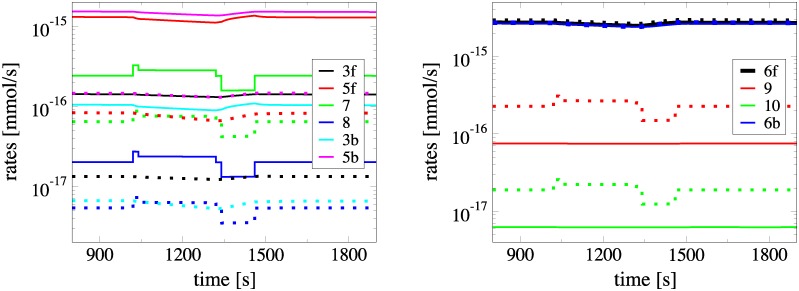
The rates related to the consumption/production of *Glc*^*a*^ and *Glc*^*n*^. Evolution of the rates related to the consumption/production of *Glc*^*a*^ (left) and *Glc*^*n*^ (right) in the ANLS (solid) and NALS (dotted) cases.

It was shown that the shift in the *Glc* to *Lac* activity along the *Glc*-path from the astrocytes to neurons, in the NALS case relative to the ANLS case is reflected in both the fast and slow dynamics. This shift is accompanied by a weakening of the *Glc*^*a*^-consuming/producing reactions 3*f*, 3*b*, 5*f*, 5*b*, 7 and 8 and a strengthening of the *Glc*^*n*^-consuming reactions 9 and 10, as shown in Fig 10. In the same figure it is shown that in the NALS case the glycolytic rate in astrocytes (*R*^7^) decreases and the glycolytic rate in neurons (*R*^9^) increases, relative to the ANLS case, supporting the findings reported previously on the influence of these reactions in coupling the oxidative rates in astrocytes and neurons (*R*^17^ and *R*^18^) with the *Lac* inflow rate in the serum.

The large *Lac* concentrations in the serum considered in the context of the exercise conditions, were shown to affect the flow of *Lac*, but left unaffected the conditions under which the *Glc* concentrations in astrocytes and neurons are coupled directly with that in the serum, since the *Glc*-path is decoupled from the *Lac*-path in the model considered. In particular, it was shown that under exercise conditions the increased rate of *Lac* diffusion from basal lamina to interstitium modified the rate of *Lac* transport between astrocytes and neurons; i.e., in the ANLS case contributed to the increased transport to neurons and the decreased transport from the astrocytes and in the NALS case contributed to the increased transport to astrocytes and the decreased transport from neurons. It is noted here that although there were significant changes in the initial conditions and the values of some parameters in the cases of normal and exercise conditions, the basic dynamical features were not altered. Specifically, there were not changes recorded in the reactions contributing to the time scales and the emerging constraints, the variables related to the modes and the number of exhausted modes. Only moderate changes were noticed on the degree to which some reactions contributed to the constraints and to the evolution of the system within these constraints.

The findings reported here are supported by experimental results in the literature. The high glycolytic rate of astrocytes (*R*^7^) in the ANLS case has been detected experimentally [4, 16, 69, 135, 136], since neurons cannot afford to sustain high glycolytic rate (*R*^9^) [6, 16]. In addition, the dominance of the glycolytic uptake in neurons (*R*^9^) has also been detected in the NALS case during neuronal activation [13–15]. Here, the dominance of *R*^7^ over *R*^9^ in the ANLS case and of *R*^9^ over *R*^7^ in the NALS case, was identified both in the fast dynamics (i.e., in the generated constraints) and the slow dynamics (i.e., in the reactions that drive the system within these constraints).

The results reported in Tables 3, 4 and 5, regarding the *TPI* and *II* indices, show that the *Lac* oxidative metabolism in neurons (*R*^18^) has a larger impact than the oxidative metabolism in astrocytes (*R*^17^) in both the ANLS and NALS cases. This is consistent with the experimental finding that the *Lac* oxidative metabolism in neurons is more important than the respective metabolism in astrocytes [77, 135]. In addition, the identifications reported here in Table 5, show that the concentration of *Lac*^*a*,*n*^ is, among others, determined by the rates *R*^7^ and *R*^9^, which act towards increasing the rate of change of *Lac*^*a*,*n*^. These rates have been reported to increase during neuronal activation in both ANLS (only *R*^7^) [16–18] and NALS (both *R*^7^ and *R*^9^) [19–21] cases, resulting in increasing *Lac*^*a*,*n*^ concentrations. This result is agreement with the elevated lactate levels that have been reported during neuronal activation [35, 137]. This feature has also been characterised as a moderate to slow process [12], which is also in agreement with our results, due to the aforementioned identifications in the slow modes of the system.

At this point it must be emphasized that the model employed here neglects metabolic processes which are currently considered important. Among them are (i) formation of pyruvate from glucose, (ii) oxidative phosphorylation in mitochondria, (iii) TCA cycle and (iv) consumption/production of ATP and NADH [8, 22, 88]. Clearly, it is possible that consideration of these processes might modify (i) the processes that are mainly responsible for the generation of the fast and slow dynamics and (ii) the conditions for which ANLS or NALS manifest. The degree to which these additional processes influence the response of the system, as it was examined here by analyzing algorithmically the fast and slow dynamics, is currently under investigation.

Finally, the ease by which the CSP tools lead to the determination of the factors that promote the development of ANLS or NALS must be highlighted. CSP allowed for these identifications by identifying the components of the model that are responsible for the fast and the slow dynamics, which are responsible for the development of the constraints in phase space (the fast) and for driving the system within these constraints (the slow).

## Conclusions

A computational model describing the evolution of the brain lactate metabolism under neuronal activation was investigated using algorithmic tools of asymptotic analysis. The model introduced in *Simpson et al*. [9] and modified by *Mangia et al*. [10] was considered, which conforms to both the ANLS and NALS hypotheses. The analysis was carried out using the CSP method, which provides algorithmic tools for the analysis of multi-scale systems, so it is not hindered by the complexity and/or the size of the system under investigation and its tools can deliver systems-level understanding. CSP was employed in order to: (i) investigate the fast/slow dynamics of the system and the metabolic profiles of neurons and astrocytes during neuronal activation and (ii) compare the dynamics of the ANLS and NALS hypotheses, under normal and exercise conditions.

It was shown that the ANLS or NALS configuration develops depending on whether the *Glc* rate of transport from astrocytes to interstitium (*R*^5^) contributes or not to the generated constraints and the fast dynamics. In the ANLS case *R*^5^ participates in the equilibria of the 4th and 5th modes, which allow *Glc* in the astrocytes and neurons to be coupled directly with that in the serum. *Lac* formation from *Glc* is more intense in astrocytes (*R*^7^) than in neurons (*R*^9^), so that the typical for the ANLS case flow of *Lac* from astrocytes to neurons is generated. In the NALS case the participation of *R*^5^ to the equilibria of the 4th and 5th modes is significantly diminished, so that only *Glc* in neurons is coupled directly with that in the serum. Therefore, *Lac* formation from *Glc* is mainly taking place in neurons (*R*^9^), so that the typical for the NALS case flow of *Lac* from neurons to astrocytes is generated.

As demonstrated, CSP is not hindered by the size or the complexity of the mechanism under investigation, therefore more complicated and larger models of brain metabolism can be considered. In many *Central Nervous System* pathogenies (like Parkinson’s and Alzheimer’s diseases) or in metabolism-related diseases (e.g., diabetes), where the degeneration of the brain structures causes abnormalities in brain function, glucose and lactate metabolism is altered; see for example [7, 138, 139]. CSP could be effectively employed in order to identify optimal strategies for the control of the system towards desired outcomes, contributing thus to the development of therapeutic treatments.

## Supporting information

S1 Text The brain lactate metabolism model. Description of the model, the initial configuration and the proper modifications.
(PDF)

S2 Text The computational singular perturbation methodology. Description of the method and it’s algorithmic tools.
(PDF)
