# Supplementary material for: Computational singular perturbation analysis of brain lactate metabolism
Source: PLoS One. 2019 Dec 17;14(12):e0226094. doi: 10.1371/journal.pone.0226094 (PMC6917278; doi:10.1371/journal.pone.0226094)
Supplement: S1 Text — Description of the model, the initial configuration and the proper modifications. (PDF) [file pone.0226094.s001.pdf]

S1 Text. The brain lactate metabolism model.

Description of the mathematical model and ANLS/NALS configurations.

The mathematical model

The brain lactate metabolic network is analyzed utilizing the model introduced in *Simpson et al.* [1] and slightly modified by *Mangia et al.* [2], which simulates the kinetic behavior of glucose and lactate among the cerebral compartments. In particular, as shown in Fig. 1 the model consists of 5 compartments: the endothelium (e), the basal lamina (bl), the astrocyte (a), the interstitium (int) and the neuron (n). Glucose (Glc) and lactate (Lac), which are the only chemical species that are accounted for in the model, are provided through the serum to the system and they cross through the various compartments, as indicated in Fig. 1.

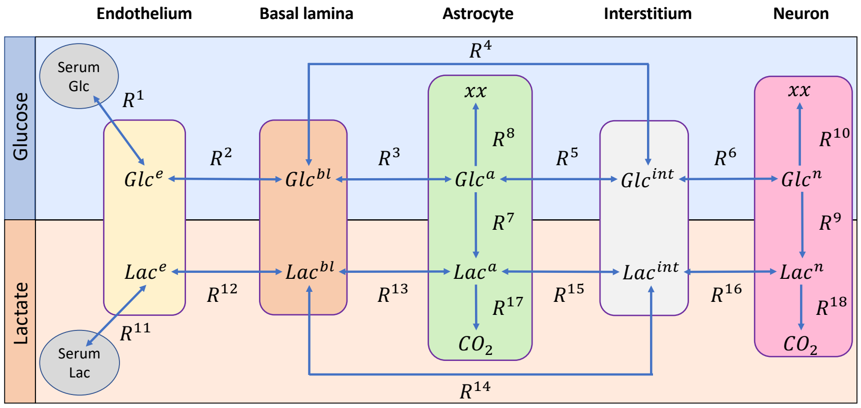

Fig 1. The 5-compartmental model introduced in [1]. The initial quantity of Glc and Lac is provided to the system through serum and then Glc^i and Lac^i transports, diffuses or metabolizes through the various compartments, ($i = e,\ bl,\ a,\ int,\ n$), by the 12 reversible and the 6 irreversible reactions.

The flow of glucose and lactate is of three types, (i) transport phenomena between two sequential compartments (reactions 1, 2, 3, 5 and 6 for glucose and 11, 12, 13, 15 and 16 for lactate), (ii) diffusion phenomena between basal lamina (bl) and interstitium (int) (reaction 4 for glucose and 14 for lactate) and (iii) metabolic inter-compartmental reactions following Michelis-Menten kinetics (reactions 7, 8, 9, 10, 17 and 18). The transport and diffusion phenomena are modelled by 12 reversible reactions, while the metabolic inter-compartmental kinetics by 6 irreversible ones. Considering that each reversible reaction R^k can be considered as two unidirectional reactions $R^{k,f}$ and $R^{k,b}$, the mathematical model can be cast in the form of Eq. (1) of the main text. The 10-dim. vector of the variables is:

$$\mathbf{y} = \begin{bmatrix} y^1 & \dots & y^{10} \end{bmatrix}^T = \begin{bmatrix} Glc^i & \dots & Lac^i & \dots \end{bmatrix}^T \tag{1}$$

where $i = e,\ bl,\ a,\ int,\ n$ denotes the compartment. The $K = 12 \times 2 + 6 = 30$ stoichiometric $\mathbf{S}_k$ vectors are:

$$
\mathbf{S}_{1f} = -\mathbf{S}_{1b} = \begin{bmatrix} +1 \\ 0 \\ 0 \\ 0 \\ 0 \\ 0 \\ 0 \\ 0 \\ 0 \\ 0 \end{bmatrix}
\mathbf{S}_{2f} = -\mathbf{S}_{2b} = \begin{bmatrix} -1 \\ +1 \\ 0 \\ 0 \\ 0 \\ 0 \\ 0 \\ 0 \\ 0 \\ 0 \end{bmatrix}
\mathbf{S}_{3f} = -\mathbf{S}_{3b} = \begin{bmatrix} 0 \\ -1 \\ +1 \\ 0 \\ 0 \\ 0 \\ 0 \\ 0 \\ 0 \\ 0 \end{bmatrix}
\mathbf{S}_{4f} = -\mathbf{S}_{4b} = \begin{bmatrix} 0 \\ -1 \\ 0 \\ +1 \\ 0 \\ 0 \\ 0 \\ 0 \\ 0 \\ 0 \end{bmatrix}
\mathbf{S}_{5f} = -\mathbf{S}_{5b} = \begin{bmatrix} 0 \\ 0 \\ -1 \\ +1 \\ 0 \\ 0 \\ 0 \\ 0 \\ 0 \\ 0 \end{bmatrix}
\mathbf{S}_{6f} = -\mathbf{S}_{6b} = \begin{bmatrix} 0 \\ 0 \\ 0 \\ -1 \\ +1 \\ 0 \\ 0 \\ 0 \\ 0 \\ 0 \end{bmatrix}
$$

$$
\mathbf{S}_{7} = \begin{bmatrix} 0 \\ 0 \\ -1 \\ 0 \\ 0 \\ 0 \\ 0 \\ +2 \\ 0 \\ 0 \end{bmatrix}
\mathbf{S}_{8} = \begin{bmatrix} 0 \\ 0 \\ -1 \\ 0 \\ 0 \\ 0 \\ 0 \\ 0 \\ 0 \\ 0 \end{bmatrix}
\mathbf{S}_{9} = \begin{bmatrix} 0 \\ 0 \\ 0 \\ 0 \\ -1 \\ 0 \\ 0 \\ 0 \\ 0 \\ +2 \end{bmatrix}
\mathbf{S}_{10} = \begin{bmatrix} 0 \\ 0 \\ 0 \\ 0 \\ -1 \\ 0 \\ 0 \\ 0 \\ 0 \\ 0 \end{bmatrix}
\mathbf{S}_{11f} = -\mathbf{S}_{11b} = \begin{bmatrix} 0 \\ 0 \\ 0 \\ 0 \\ 0 \\ +1 \\ 0 \\ 0 \\ 0 \\ 0 \end{bmatrix}
\mathbf{S}_{12f} = -\mathbf{S}_{12b} = \begin{bmatrix} 0 \\ 0 \\ 0 \\ 0 \\ 0 \\ -1 \\ +1 \\ 0 \\ 0 \\ 0 \end{bmatrix}
\mathbf{S}_{13f} = -\mathbf{S}_{13b} = \begin{bmatrix} 0 \\ 0 \\ 0 \\ 0 \\ 0 \\ 0 \\ -1 \\ +1 \\ 0 \\ 0 \end{bmatrix}
$$

$$
\mathbf{S}_{14f} = -\mathbf{S}_{14b} = \begin{bmatrix} 0 \\ 0 \\ 0 \\ 0 \\ 0 \\ 0 \\ -1 \\ 0 \\ +1 \\ 0 \end{bmatrix}
\mathbf{S}_{15f} = -\mathbf{S}_{15b} = \begin{bmatrix} 0 \\ 0 \\ 0 \\ 0 \\ 0 \\ 0 \\ 0 \\ -1 \\ +1 \\ 0 \end{bmatrix}
\mathbf{S}_{16f} = -\mathbf{S}_{16b} = \begin{bmatrix} 0 \\ 0 \\ 0 \\ 0 \\ 0 \\ 0 \\ 0 \\ 0 \\ -1 \\ +1 \end{bmatrix}
\mathbf{S}_{17} = \begin{bmatrix} 0 \\ 0 \\ 0 \\ 0 \\ 0 \\ 0 \\ 0 \\ -1 \\ 0 \\ 0 \end{bmatrix}
\mathbf{S}_{18} = \begin{bmatrix} 0 \\ 0 \\ 0 \\ 0 \\ 0 \\ 0 \\ 0 \\ 0 \\ 0 \\ -1 \end{bmatrix}
\tag{2}
$$

	Forward rate	Backward rate
R^1	$\dfrac{Glc^s\left(K+\frac{Glc^e}{V^e}\right)}{K^2R_{oo}+KR_{oi}Glc^s+KR_{io}\frac{Glc^e}{V^e}+R_{ee}\frac{Glc^e}{V^e}Glc^s}$	$\dfrac{\frac{Glc^e}{V^e}\left(K+Glc^s\right)}{K^2R_{oo}+KR_{oi}Glc^s+KR_{io}\frac{Glc^e}{V^e}+R_{ee}\frac{Glc^e}{V^e}Glc^s}$
R^2	$\dfrac{\frac{Glc^e}{V^e}\left(K+\frac{Glc^{bl}}{V^{bl}}\right)}{K^2R_{oo}+KR_{oi}\frac{Glc^{bl}}{V^{bl}}+KR_{io}\frac{Glc^e}{V^e}+R_{ee}\frac{Glc^e}{V^e}\frac{Glc^{bl}}{V^{bl}}}$	$\dfrac{\frac{Glc^{bl}}{V^{bl}}\left(K+\frac{Glc^e}{V^e}\right)}{K^2R_{oo}+KR_{oi}\frac{Glc^{bl}}{V^{bl}}+KR_{io}\frac{Glc^e}{V^e}+R_{ee}\frac{Glc^e}{V^e}\frac{Glc^{bl}}{V^{bl}}}$
R^3	$\dfrac{\frac{Glc^{bl}}{V^{bl}}\left({}^{a}K+\frac{Glc^a}{V^a}\right)}{{}^{a}K^{2\,bla}R_{oo}+{}^{a}K^{bla}R_{oi}\frac{Glc^{bl}}{V^{bl}}+{}^{a}K^{bla}R_{io}\frac{Glc^a}{V^a}+{}^{bla}R_{ee}\frac{Glc^a}{V^a}\frac{Glc^{bl}}{V^{bl}}}$	$\dfrac{\frac{Glc^a}{V^a}\left({}^{a}K+\frac{Glc^{bl}}{V^{bl}}\right)}{{}^{a}K^{2\,bla}R_{oo}+{}^{a}K^{bla}R_{oi}\frac{Glc^{bl}}{V^{bl}}+{}^{a}K^{bla}R_{io}\frac{Glc^a}{V^a}+{}^{bla}R_{ee}\frac{Glc^a}{V^a}\frac{Glc^{bl}}{V^{bl}}}$
R^5	$\dfrac{\frac{Glc^a}{V^a}\left({}^{a}K+\frac{Glc^{int}}{V^{int}}\right)}{{}^{a}K^{2\,inta}R_{oo}+{}^{a}K^{inta}R_{oi}\frac{Glc^{int}}{V^{int}}+{}^{a}K^{inta}R_{io}\frac{Glc^a}{V^a}+{}^{inta}R_{ee}\frac{Glc^a}{V^a}\frac{Glc^{int}}{V^{int}}}$	$\dfrac{\frac{Glc^{int}}{V^{int}}\left({}^{a}K+\frac{Glc^a}{V^a}\right)}{{}^{a}K^{2\,inta}R_{oo}+{}^{a}K^{inta}R_{oi}\frac{Glc^{int}}{V^{int}}+{}^{a}K^{inta}R_{io}\frac{Glc^a}{V^a}+{}^{inta}R_{ee}\frac{Glc^a}{V^a}\frac{Glc^{int}}{V^{int}}}$
R^6	$\dfrac{\frac{Glc^{int}}{V^{int}}\left({}^{n}K+\frac{Glc^n}{V^n}\right)}{{}^{n}K^{2n}R_{oo}+{}^{n}K^{n}R_{oi}\frac{Glc^{int}}{V^{int}}+{}^{n}K^{n}R_{io}\frac{Glc^n}{V^n}+{}^{n}R_{ee}\frac{Glc^n}{V^n}\frac{Glc^{int}}{V^{int}}}$	$\dfrac{\frac{Glc^n}{V^n}\left({}^{n}K+\frac{Glc^{int}}{V^{int}}\right)}{{}^{n}K^{2n}R_{oo}+{}^{n}K^{n}R_{oi}\frac{Glc^{int}}{V^{int}}+{}^{n}K^{n}R_{io}\frac{Glc^n}{V^n}+{}^{n}R_{ee}\frac{Glc^n}{V^n}\frac{Glc^{int}}{V^{int}}}$
R^{11}	$\dfrac{Lac^s\left({}^{e}K_L+\frac{Lac^e}{V^e}\right)}{{}^{e}K_L^2\,{}^{e}_{L}R_{oo}+{}^{e}K_L\,{}^{e}_{L}R_{oi}Lac^s+{}^{e}K_L\,{}^{e}_{L}R_{io}\frac{Lac^e}{V^e}+{}^{e}_{L}R_{ee}\frac{Lac^e}{V^e}Lac^s}$	$\dfrac{\frac{Lac^e}{V^e}\left({}^{e}K_L+Lac^s\right)}{{}^{e}K_L^2\,{}^{e}_{L}R_{oo}+{}^{e}K_L\,{}^{e}_{L}R_{oi}Lac^s+{}^{e}K_L\,{}^{e}_{L}R_{io}\frac{Lac^e}{V^e}+{}^{e}_{L}R_{ee}\frac{Lac^e}{V^e}Lac^s}$
R^{12}	$\dfrac{\frac{Lac^e}{V^e}\left({}^{e}K_L+\frac{Lac^{bl}}{V^{bl}}\right)}{{}^{e}K_L^2\,{}^{e}_{L}R_{oo}+{}^{e}K_L\,{}^{e}_{L}R_{oi}\frac{Lac^{bl}}{V^{bl}}+{}^{e}K_L\,{}^{e}_{L}R_{io}\frac{Lac^e}{V^e}+{}^{e}_{L}R_{ee}\frac{Lac^e}{V^e}\frac{Lac^{bl}}{V^{bl}}}$	$\dfrac{\frac{Lac^{bl}}{V^{bl}}\left({}^{e}K_L+\frac{Lac^e}{V^e}\right)}{{}^{e}K_L^2\,{}^{e}_{L}R_{oo}+{}^{e}K_L\,{}^{e}_{L}R_{oi}\frac{Lac^{bl}}{V^{bl}}+{}^{e}K_L\,{}^{e}_{L}R_{io}\frac{Lac^e}{V^e}+{}^{e}_{L}R_{ee}\frac{Lac^e}{V^e}\frac{Lac^{bl}}{V^{bl}}}$
R^{13}	$\dfrac{\frac{Lac^{bl}}{V^{bl}}\left({}^{a}K_L+\frac{Lac^a}{V^a}\right)}{{}^{a}K_L^2\,{}^{bla}_{L}R_{oo}+{}^{a}K_L\,{}^{bla}_{L}R_{oi}\frac{Lac^{bl}}{V^{bl}}+{}^{a}K_L\,{}^{bla}_{L}R_{io}\frac{Lac^a}{V^a}+{}^{bla}_{L}R_{ee}\frac{Lac^a}{V^a}\frac{Lac^{bl}}{V^{bl}}}$	$\dfrac{\frac{Lac^a}{V^a}\left({}^{a}K_L+\frac{Lac^{bl}}{V^{bl}}\right)}{{}^{a}K_L^2\,{}^{bla}_{L}R_{oo}+{}^{a}K_L\,{}^{bla}_{L}R_{oi}\frac{Lac^{bl}}{V^{bl}}+{}^{a}K_L\,{}^{bla}_{L}R_{io}\frac{Lac^a}{V^a}+{}^{bla}_{L}R_{ee}\frac{Lac^a}{V^a}\frac{Lac^{bl}}{V^{bl}}}$
R^{15}	$\dfrac{\frac{Lac^a}{V^a}\left({}^{a}K_L+\frac{Lac^{int}}{V^{int}}\right)}{{}^{a}K_L^2\,{}^{inta}_{L}R_{oo}+{}^{a}K_L\,{}^{inta}_{L}R_{oi}\frac{Lac^{int}}{V^{int}}+{}^{a}K_L\,{}^{inta}_{L}R_{io}\frac{Lac^a}{V^a}+{}^{inta}_{L}R_{ee}\frac{Lac^a}{V^a}\frac{Lac^{int}}{V^{int}}}$	$\dfrac{\frac{Lac^{int}}{V^{int}}\left({}^{a}K_L+\frac{Lac^a}{V^a}\right)}{{}^{a}K_L^2\,{}^{inta}_{L}R_{oo}+{}^{a}K_L\,{}^{inta}_{L}R_{oi}\frac{Lac^{int}}{V^{int}}+{}^{a}K_L\,{}^{inta}_{L}R_{io}\frac{Lac^a}{V^a}+{}^{inta}_{L}R_{ee}\frac{Lac^a}{V^a}\frac{Lac^{int}}{V^{int}}}$
R^{16}	$\dfrac{\frac{Lac^{int}}{V^{int}}\left({}^{n}K_L+\frac{Lac^n}{V^n}\right)}{{}^{n}K_L^2\,{}^{n}_{L}R_{oo}+{}^{n}K_L\,{}^{n}_{L}R_{oi}\frac{Lac^{int}}{V^{int}}+{}^{n}K_L\,{}^{n}_{L}R_{io}\frac{Lac^n}{V^n}+{}^{n}_{L}R_{ee}\frac{Lac^n}{V^n}\frac{Lac^{int}}{V^{int}}}$	$\dfrac{\frac{Lac^n}{V^n}\left({}^{n}K_L+\frac{Lac^{int}}{V^{int}}\right)}{{}^{n}K_L^2\,{}^{n}_{L}R_{oo}+{}^{n}K_L\,{}^{n}_{L}R_{oi}\frac{Lac^{int}}{V^{int}}+{}^{n}K_L\,{}^{n}_{L}R_{io}\frac{Lac^n}{V^n}+{}^{n}_{L}R_{ee}\frac{Lac^n}{V^n}\frac{Lac^{int}}{V^{int}}}$

Table 1. Reaction rates for Glucose and Lactate transport through the various compartments. The first column denotes the forward rate $R^{i,f}$ and the second one the backward rate $R^{i,b}$.

for which the reactions have been considered to be unidirectional. The respective reaction rates R^k are of three types, (i) transport phenomena, (ii) diffusion phenomena and (iii) metabolic inter-compartmental Michaelis-Menten reactions.

The transport related reaction rates are shown in Table 1, in which the first column denotes the forward rates $R^{i,f}$ and the second one the backward rates $R^{i,b}$. The diffusion related reaction rates between the basal lamina (bl) compartment and the interstitium (int) one are:

$$\begin{aligned} R^{4f} &= k_{app}\frac{Glc^{bl}}{V^{bl}}10^{-15} \qquad & R^{4b} &= k_{app}\frac{Glc^{int}}{V^{bl}}10^{-15} \\ R^{14f} &= k_{app}\frac{Lac^{bl}}{V^{bl}}10^{-15} \qquad & R^{14b} &= k_{app}\frac{Lac^{int}}{V^{bl}}10^{-15} \end{aligned} \tag{3}$$

Finally, the metabolic inter-compartmental Michaelis-Menten reactions rates are:

$$\begin{aligned} R^{7} &= \frac{{}^{a}V_h\frac{Glc^a}{V^a}}{K_h+\frac{Glc^a}{V^a}} \qquad & R^{8} &= \frac{1/12\,{}^{a}V_h\frac{Glc^a}{V^a}}{K_h+\frac{Glc^a}{V^a}} \qquad & R^{9} &= \frac{{}^{n}V_h\frac{Glc^n}{V^n}}{K_h+\frac{Glc^n}{V^n}} \\ R^{10} &= \frac{1/12\,{}^{n}V_h\frac{Glc^n}{V^n}}{K_h+\frac{Glc^n}{V^n}} \qquad & R^{17} &= \frac{{}^{a}V_L\frac{Lac^a}{V^a}}{K_L+\frac{Lac^a}{V^a}} \qquad & R^{18} &= \frac{{}^{n}V_L\frac{Lac^n}{V^n}}{K_L+\frac{Lac^n}{V^n}} \end{aligned} \tag{4}$$

Equations in Table 1 along with Eq. (3) and (4) consist the overall R^k, which when left multiplied by the column vectors $\mathbf{S}_k$ in Eq. (2) form the vector field $\mathbf{g}(\mathbf{y})$, according to Eq. (1) of the main text.

The ANLS/NALS parameter configuration

When the original model was introduced in [1], two configurations were proposed for the model, one appropriate for describing the ANLS hypothesis and one for the NALS one. Both configurations have been considered not only for the enhancement of the model under specific conditions [2], but also for the construction of more complicated models [3, 4]. In this work, the configurations introduced in *Mangia et al.* for both ANLS and NALS hypotheses were adopted, due to the fitting of the model to *in vivo* data obtained with fMRS in humans [2].

Except from the parameter fitting, the configurations utilized by *Mangia et al.* are slightly different than the ones used in [1]. However, we were able to reproduce the findings in *Mangia et al.* for both hypotheses and display them in Fig. 2. In particular, Figure 2 shows the qualitatively different response of *Lac* flow, when the ANLS or NALS configuration is adopted. In the ANLS case, the *Lac* flow follows the direction astrocytes-interstitium-neurons, which is depicted in Fig. 2 by the positive values of R^{15} (astrocytes to interstitium) and R^{16} (interstitium to neurons). On the other hand, in the NALS case the *Lac* flow follows the opposite direction neurons-interstitium-astrocytes, which is depicted in Fig. 2 by the negative values of R^{15} and R^{16}. These findings are in exact agreement with the results presented in the *Mangia et al.* paper (see Fig. 3b of [2]).

The results in *Mangia et al.* were able to be reproduced with great accuracy for both the ANLS and NALS cases, after contacting with the authors [2]. The simulations and the analysis that followed were carried out for the ANLS case, for which the parameter configuration is displayed in Table 2 and for the NALS case, for which only the differences from the ANLS configuration are reported in Table 3. When shifting from ANLS to NALS configuration, the parameter alterations reflect on the rate changes that are shown in Fig. 3, so that:

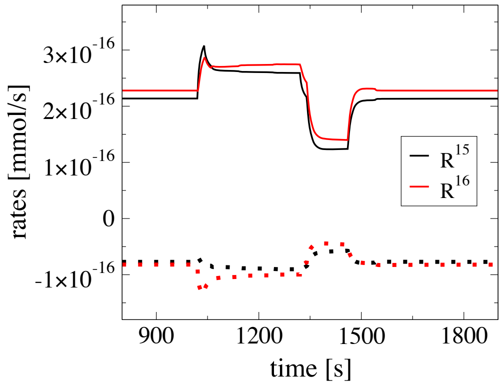

Fig 2. Comparison of the ANLS model (solid lines) with the NALS one (dotted lines). The Lac flow from astrocyte to interstitium (R^{15}) and from interstitium to neuron (R^{16}).

- the increase of the astrocytic Glc transport (${}^{bla}R_{xx}$ and ${}^{inta}R_{xx}$, where $xx = oo, oi, io, ee$) causes the decrease of both R^3 and R^5 rates, as shown by the respective expressions in Table 1,
- the decrease of the ${}^aV_h$ capacity causes the decrease of R^7 and R^8, as shown by the respective expressions in Eq. (4),
- the increase of the ${}^nV_h$ capacity causes the increase of R^9 and R^{10}, as shown by the respective expressions in Eq. (4) and
- the minor decrease of the ${}^aV_L$ and ${}^nV_L$ capacities causes the minor decrease of R^{17} and R^{18}, respectively, as shown by the respective expressions in Eq. (4).

All the other parameters remain the same and thus, the rates they relate to do not have major alterations, when shifting from ANLS to NALS case, as shown in Fig. 3.

Finally, it should be noted that the Michaelis-Menten maximum velocities (capacities) ${}^aV_h$, ${}^aV_L$, ${}^nV_h$ and ${}^nV_L$ are the parameters changing during simulation to simulate neuronal activation. These changes are the proportional for both ANLS and NALS configurations, except from ${}^nV_h$, which is assumed constant in the ANLS case. The initial condition for the simulation is $Glc^s = 5.5\ mmol/L$ and $Lac^s = 1\ mmol/L$.

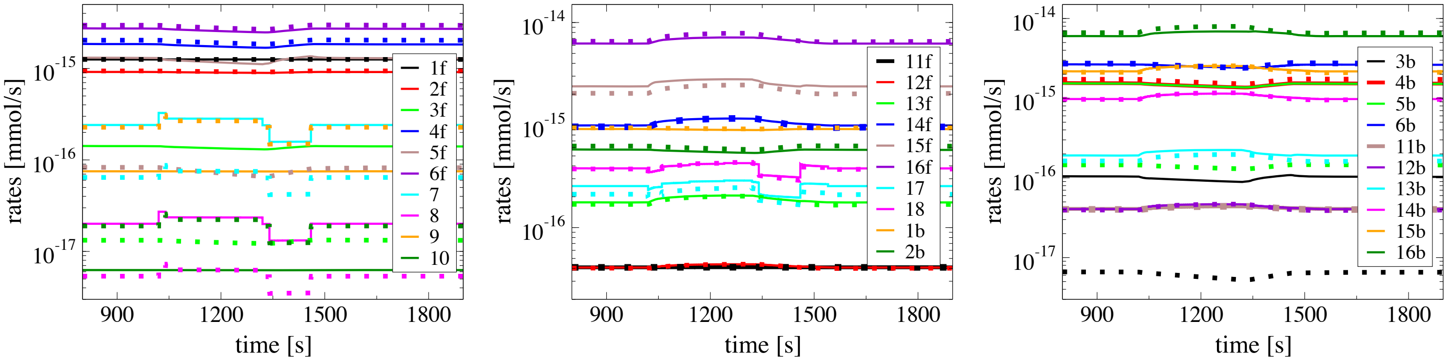

Fig 3. Evolution of the all the rates involved in the mathematical model, as presented in Table 1 and Eqs. (3, 4) in the ANLS (solid) and NALS (dotted) cases.

**Volumes** $(\times 10^{-15})$
$V^e = 1.2852$, $V^{bl} = 1.02$, $V^a = 19.72$, $V^{int} = 12.24$, $V^n = 33.66$
**Diffusion constant**
$k_{app} = 1$
**Endothelial** ***Glc*** **transport**
$R_{oi} = R_{io} = R_{ee} = 32.5 \times 10^{13}$, $R_{oo} = R_{io} + R_{oi} - R_{ee}$, $K = 8$
**Astrocyte** ***Glc*** **transport (basal lamina to astrocyte)**
${}^{bla}R_{oi} = 13.75 \times 10^{14}$, ${}^{bla}R_{io} = {}^{bla}R_{ee} = 1 \times 10^{15}$, ${}^{bla}R_{oo} = {}^{bla}R_{io} + {}^{bla}R_{oi} - {}^{bla}R_{ee}$, ${}^{a}K = 8$
**Astrocyte** ***Glc*** **transport (astrocyte to interstitium)**
${}^{inta}R_{oi} = 11 \times 10^{13}$, ${}^{inta}R_{io} = {}^{inta}R_{ee} = 8 \times 10^{13}$, ${}^{inta}R_{oo} = {}^{inta}R_{io} + {}^{inta}R_{oi} - {}^{inta}R_{ee}$
**Neuron** ***Glc*** **transport**
${}^{n}R_{oi} = 11 \times 10^{13}$, ${}^{n}R_{io} = {}^{n}R_{ee} = 8 \times 10^{13}$, ${}^{n}R_{oo} = {}^{n}R_{io} + {}^{n}R_{oi} - {}^{n}R_{ee}$, ${}^{n}K = 4$
**Endothelial lactate transport**
${}^{e}_{L}R_{oi} = 2.8 \times 10^{15}$, ${}^{e}_{L}R_{io} = {}^{e}_{L}R_{ee} = 2 \times 10^{13}$, ${}^{e}_{L}R_{oo} = {}^{e}_{L}R_{io} + {}^{e}_{L}R_{oi} - {}^{e}_{L}R_{ee}$, ${}^{e}K_L = 8$
**Astrocyte** ***Lac*** **transport (basal lamina to astrocyte)**
${}^{bla}_{L}R_{oi} = 8.25 \times 10^{14}$, ${}^{bla}_{L}R_{io} = 4.125 \times 10^{14}$, ${}^{bla}_{L}R_{ee} = 1.125 \times 10^{14}$, ${}^{bla}_{L}R_{oo} = {}^{bla}_{L}R_{io} + {}^{bla}_{L}R_{oi} - {}^{bla}_{L}R_{ee}$, ${}^{a}K_L = 5$
**Astrocyte** ***Lac*** **transport (astrocyte to interstitium)**
${}^{inta}_{L}R_{oi} = 6.6 \times 10^{13}$, ${}^{inta}_{L}R_{io} = 3.3 \times 10^{13}$, ${}^{inta}_{L}R_{ee} = 1 \times 10^{13}$, ${}^{inta}_{L}R_{oo} = {}^{inta}_{L}R_{io} + {}^{inta}_{L}R_{oi} - {}^{inta}_{L}R_{ee}$
**Neuron** ***Lac*** **transport**
${}^{n}_{L}R_{oi} = 2 \times 10^{14}$, ${}^{n}_{L}R_{io} = {}^{n}_{L}R_{ee} = 1 \times 10^{13}$, ${}^{n}_{L}R_{oo} = {}^{n}_{L}R_{io} + {}^{n}_{L}R_{oi} - {}^{n}_{L}R_{ee}$, ${}^{n}K_L = 0.7$
**Metabolic constants**
$K_h = 0.045$, $K_L = 2$, ${}^{a}V_h = 2.49 \times 10^{-16}$, ${}^{n}V_h = 7.73 \times 10^{-17}$, ${}^{a}V_L = 7.19 \times 10^{-16}$, ${}^{n}V_L = 1.22 \times 10^{-15}$

Table 2. ANLS parameter configuration, adopted by *Mangia et al.*, [2]. The units of the parameters are: volumes V in L, diffusion constant in s^{-1}, R terms in $s/mmol$, K constants in $mmol/L$ and Michaelis-Menten V (capacity) in $mmol/s$.

**Astrocyte** ***Glc*** **transport (basal lamina to astrocyte)**
${}^{bla}R_{oi} = 15.62 \times 10^{15}$, ${}^{bla}R_{io} = {}^{bla}R_{ee} = 12.5 \times 10^{15}$, ${}^{bla}R_{oo} = {}^{bla}R_{io} + {}^{bla}R_{oi} - {}^{bla}R_{ee}$
**Astrocyte** ***Glc*** **transport (astrocyte to interstitium)**
${}^{inta}R_{oi} = 12.5 \times 10^{14}$, ${}^{inta}R_{io} = {}^{inta}R_{ee} = 9.5 \times 10^{14}$, ${}^{inta}R_{oo} = {}^{inta}R_{io} + {}^{inta}R_{oi} - {}^{inta}R_{ee}$
**Metabolic constants**
${}^{a}V_h = 6.76 \times 10^{-17}$, ${}^{n}V_h = 2.34 \times 10^{-16}$, ${}^{a}V_L = 6.67 \times 10^{-16}$, ${}^{n}V_L = 1.12 \times 10^{-15}$

Table 3. NALS parameter configuration (only differences from ANLS case), adopted by *Mangia et al.*, [2]. The units of the parameters as in Table 2

References

1. Simpson IA, Carruthers A, Vannucci SJ. Supply and demand in cerebral energy metabolism: the role of nutrient transporters. J Cereb Blood Flow Metab. 2007;27(11):1766–91.

2. Mangia S, Simpson IA, Vannucci SJ, Carruthers A. The in vivo neuron-to-astrocyte lactate shuttle in human brain: evidence from modeling of measured lactate levels during visual stimulation. Journal of Neurochemistry. 2009;109:55–62.

3. DiNuzzo M, Mangia S, Maraviglia B, Giove F. Changes in glucose uptake rather than lactate shuttle take center stage in subserving neuroenergetics: evidence from mathematical modeling. Journal of Cerebral Blood Flow & Metabolism. 2010;30(3):586–602.

4. Cloutier M, Bolger FB, Lowry JP, Wellstead P. An integrative dynamic model of brain energy metabolism using in vivo neurochemical measurements. Journal of

computational neuroscience. 2009;27(3):391.
