# Supplementary material for: Computational singular perturbation analysis of brain lactate metabolism
Source: PLoS One. 2019 Dec 17;14(12):e0226094. doi: 10.1371/journal.pone.0226094 (PMC6917278; doi:10.1371/journal.pone.0226094)
Supplement: S2 Text — Description of the method and it’s algorithmic tools. (PDF) [file pone.0226094.s002.pdf]

S2 Text. The Computational Singular Perturbation methodology Description of the method and it's algorithmic tools.

The Computational Singular Perturbation methodology and it's algorithmic tools

The evolution of the chemical species among the cellular compartments of a brain metabolic network can be often studied with the utilization of point mathematical models. As a result, the time evolution of the concentrations of the metabolites can be determined by a system of Ordinary Differential Equations (ODEs):

$$\frac{d\mathbf{y}}{dt} = \sum_{k=1}^{K} \mathbf{S}_k R^k = \mathbf{g}(\mathbf{y}) \tag{1}$$

where $\mathbf{y}$ is the N-dim. column vector containing the concentrations of the metabolites at the various compartments, $\mathbf{S}_k$ denotes the N-dim. stoichiometric vector of each of the K reactions and R^k is the related reaction rate.

According to CSP, the system in Eq. (1) can be cast into the following form [1, 2]:

$$\frac{d\mathbf{y}}{dt} = \mathbf{g}(\mathbf{y}) = \sum_{n=1}^{N} \mathbf{a}_n f^n \tag{2}$$

where $\mathbf{a}_n$ is the N-dim. CSP column basis vector of the n-th mode and f^n is the related amplitude:

$$f^n = \mathbf{b}^n \cdot \mathbf{g}(\mathbf{y}) = \sum_{k=1}^{K} \left(\mathbf{b}^n \cdot \mathbf{S}_k\right) R^k \tag{3}$$

where $\mathbf{b}^i$ is the N-dim. dual row vector that satisfies the orthogonality condition $\mathbf{b}^i \cdot \mathbf{a}_j = \delta^i_j$ [1, 3]. The amplitude f^n of the n-th mode provides a measure of the projection of the vector field $\mathbf{g}(\mathbf{y})$ on the CSP vector $\mathbf{a}_n$. Therefore, all amplitudes are set positive, by properly adjusting the sign of the N-dim. row vectors $\mathbf{b}^n$ (and therefore the sign of $\mathbf{a}_n$, so that the orthogonality conditions are preserved) [1, 2].

When the system in Eq. (2) exhibits M time scales that are (i) of dissipative nature, i.e. the components of the system that generate them tend to drive the system towards equilibrium and (ii) much faster than the rest, Eq. (2) can be cast into the following form:

$$\frac{d\mathbf{y}}{dt} = \mathbf{g}_{fast}(\mathbf{y}) + \mathbf{g}_{slow}(\mathbf{y}) = \sum_{r=1}^{M} \mathbf{a}_r f^r + \sum_{s=M+1}^{N} \mathbf{a}_s f^s \tag{4}$$

The terms $\mathbf{g}_{fast}(\mathbf{y}) = \sum_{r=1}^{M} \mathbf{a}_r f^r$ (fast modes) and $\mathbf{g}_{slow} = \sum_{s=M+1}^{N} \mathbf{a}_s f^s$ (slow modes) relate to the M fast and $N - M$ slow, respectively, time scales. When the fast dissipative time scales become exhausted, the corresponding amplitudes become negligibly small and a reduced model is constructed as:

$$f^r \approx 0 \quad (r = 1, \ldots, M) \qquad\qquad \frac{d\mathbf{y}}{dt} \approx \sum_{s=M+1}^{N} \mathbf{a}_s f^s \tag{5}$$

The first of these two relations is an M-dim. system of algebraic equations and defines an $N-M$-dim. manifold $\mathscr{M}$, while the second relation is an N-dim. system of ODEs that governs the slow evolution of the system on this manifold. Physically, the amplitude f^n ($n = M+1, \ldots, N$) provides a measure of the impact of the n-th slow CSP mode ($\mathbf{a}_n f^n$) to the slow evolution of the process on the manifold, i.e. on $\mathbf{g}_{slow}$. On the other hand, the time scale τ_n describes the time frame of action of the of the n-th CSP mode.

To leading order, the CSP vectors $\mathbf{a}_i$ and $\mathbf{b}^i$ ($i = 1, \ldots, N$), can be approximated by the right and left, respectively, eigenvectors of the $N \times N$-dim. Jacobian $\mathbf{J}$ of $\mathbf{g}(\mathbf{y})$ [1, 2, 4]. Therefore, since we are interested in leading order accuracy, in the following analysis the CSP basis vectors will be approximated by the eigenvectors of $\mathbf{J}$.

The time scales of the system in Eq. (2) can be approximated by the relation $\tau_n = |\lambda_n|^{-1}$ ($n = 1, \ldots, N$), where λ_n is the n-th non-zero eigenvalue of the Jacobian $\mathbf{J}$ of $\mathbf{g}(\mathbf{y})$. This eigenvalue is defined as $\lambda_n = \boldsymbol{\beta}^n \cdot \mathbf{J} \cdot \boldsymbol{\alpha}_n$, where $\boldsymbol{\alpha}_n$ and $\boldsymbol{\beta}^n$ are the n-th right (column) and left (row), respectively, eigenvectors of $\mathbf{J}$; $\boldsymbol{\beta}^i \cdot \boldsymbol{\alpha}_j = \delta^i_j$. As a result, the n-th eigenvalue can be expressed as a summation of K terms:

$$\lambda_n = \boldsymbol{\beta}^n \sum_{k=1}^{K} \nabla \left(\mathbf{S}_k R^k \right) \boldsymbol{\alpha}_n = c^n_1 + \ldots + c^n_K \tag{6}$$

since $\mathbf{J} = \nabla(\mathbf{S}_1 R^1) + \ldots + \nabla(\mathbf{S}_K R^K)$ [5–7]. For real eigenvalues (the extension to the case of complex eigenvalues is straightforward [5]), the magnitude of the terms c^n_k is indicative of the contribution of the k-th reaction to the n-th eigenvalue. The c^n_k terms can be either positive or negative. When positive (negative), it implies that the k-th reaction contributes to an explosive (dissipative) character of the n-th time scale τ_n. By definition, explosive (dissipative) time scales relate to the components of the system that tend to drive it away from (towards) equilibrium. The contribution of each of the K reactions to the n-th time scale τ_n can be assessed through the related term in Eq. (5), by employing the *Time scale Participation Index* (*TPI*):

$$J^n_k = \frac{c^n_k}{\sum_{i=1}^{K} |c^n_i|} \tag{7}$$

where $k = 1, \ldots, K$ and by definition $\sum_{k=1}^{K} |J^n_k| = 1$ [5, 6]. J^n_k measures the relative contribution of the k-th reaction to the n-th eigenvalue λ_n and, therefore, to the corresponding time scale τ_n [8].

After the calculation of all time scales, the N modes are ordered from fastest to slowest based on their associated time scales:

$$\tau_1 < \tau_2 < ... < \tau_{M-1} < \tau_M < \tau_{M+1} < ... < \tau_{N-1} < \tau_N \tag{8}$$

In order to determine M, that is the number of the fast modes, the criterion that is used is based on an error vector $\mathbf{y}_{error}$ which is generated on the basis of the state vector $\mathbf{y}$, as follows [9]:

$$y^i_{error} = \epsilon^i_{rel} y^i + \epsilon^i_{abs} \qquad\qquad i = 1, \ldots, N \tag{9}$$

where ϵ^i_{rel} and ϵ^i_{abs} are the maximum relative and absolute errors on the i-th element of the state vector y, respectively. In the current analysis, $\epsilon^i_{rel} = \epsilon = \tau_M / \tau_{M+1}$ and $\epsilon^i_{abs} = 10^{-16}$ which are considered typical values for such models under consideration. In that case, the number M of fast time scales which are considered exhausted, is defined as the largest integer (between 1 and N) which satisfies the following inequality:

$$|\tau_{M+1} \sum_{i=1}^{M} \boldsymbol{\alpha}_i f^i| < \mathbf{y}_{error} \tag{10}$$

The inequality (10) guarantees that the trajectory remains close to the manifold within specified bounds and is not diverted far from it by marching in time according to the simplified non-stiff Eq. (5).

From the definition of the amplitudes of the modes, it follows that the amplitude of the n-th exhausted mode ($n = 1, \ldots, M$) is calculated as follows:

$$f^n = \boldsymbol{\beta}^n \mathbf{g}(\mathbf{y}) = (\boldsymbol{\beta}^n \mathbf{S}_1)R^1 + \ldots + (\boldsymbol{\beta}^n \mathbf{S}_K)R^K \approx 0 \quad (11)$$

The quantity $(\boldsymbol{\beta}^n \mathbf{S}_k)R^k$ denotes the contribution of the k-th process ($k = 1, \ldots, K$) to the n-th fast amplitude. Therefore, Eq. (11) shows that the decay of the fast amplitudes to negligibly small values is the result of equilibrations developing among the various processes of the model. As a result, the *Amplitude Participation Index* (*API*) P_k^n can provide a measure of the relative contribution of the k-th reaction to the cancellations among the additive terms in the n-th algebraic relation that approximates the manifold, $f^n \approx 0$ ($n = 1, \ldots, M$):

$$P_k^n = \frac{(\boldsymbol{\beta}^n \mathbf{S}_k)R^k}{\sum_{i=1}^{K} |(\boldsymbol{\beta}^n \mathbf{S}_i)R^i|} \quad (12)$$

where by definition $\sum_{k=1}^{K} |P_k^n| = 1$, [3, 5, 6].

Furthermore, the decomposition into fast and slow modes allows for the introduction of the *slow Importance Index* (*II*) I_k^i which provides a measure of the relative importance of the k-th process to the prodution/consumption of the i-th chemical species in the context of its slow evolution on the manifold [3, 9–11]:

$$I_k^i = \frac{\sum_{s=M+1}^{N} \alpha_s^i (\boldsymbol{\beta}^s \mathbf{S}_k)R^k}{\sum_{j=1}^{K} |\sum_{s=M+1}^{N} \alpha_s^i (\boldsymbol{\beta}^s \mathbf{S}_j)R^j|} \quad (13)$$

where, by definition, $\sum_{k=1}^{K} |I_k^i| = 1$. For a given chemical species, the reactions with the largest I_k^i are the rate controlling reactions for that species, as they are the most responsible for its slow evolution on the manifold [11].

Each chemical species associates differently to each exhausted CSP mode, e.g., a chemical species can relate mostly to the n-th CSP mode but very less to the m-th mode ($n, m = 1, \ldots, M$). In order to assess the relation of the i-th mode to the various chemical species, the *CSP Pointer* (Po) has been introduced [10, 12]:

$$\mathbf{D}^i = diag\left[\boldsymbol{\alpha}_i \boldsymbol{\beta}^i\right] = \left[\alpha_i^1 \beta_1^i, \alpha_i^2 \beta_2^i, \ldots, \alpha_i^N \beta_N^i\right] \quad (14)$$

where, due to the orthogonality condition $\boldsymbol{\beta}^i \cdot \boldsymbol{\alpha}_j = \delta_j^i$, the sum of all N elements of $\mathbf{D}^i$ equals unity; i.e., $\sum_{i=1}^{N} \alpha_k^i \beta_i^k = 1$. Values of $\alpha_k^i \beta_i^k$ close to unity indicate that the i-th variable is strongly connected to k-th CSP mode and the corresponding time scale. Therefore, the i-th variable has a significant influence on the terms participating in the cancellations occurring in the k-th amplitude of the exhausted CSP mode. Chemical species with *CSP Po* values close to unity are potentially in *Quasi Steady-State* (QSS).

References

1. Lam S, Goussis D. Understanding complex chemical kinetics with computational singular perturbation. In: Symposium (International) on Combustion. vol. 22; 1989. p. 931–941.

2. Lam S, Coussis D. Conventional asymptotics and computational singular perturbation for simplified kinetics modelling. In: Reduced kinetic mechanisms and asymptotic approximations for methane-air flames; 1991. p. 227–242.

3. Lam S, Goussis D. The CSP method for simplifying kinetics. International Journal of Chemical Kinetics. 1994;26(4):461–486.

4. Tingas EA, Kyritsis DC, Goussis DA. Ignition delay control of DME/air and EtOH/air homogeneous autoignition with the use of various additives. Fuel. 2016;169:15–24.

5. Goussis DA, Najm HN. Model reduction and physical understanding of slowly oscillating processes: the circadian cycle. Multiscale Modeling & Simulation. 2006;5(4):1297–1332.

6. Goussis DA, Skevis G. Nitrogen chemistry controlling steps in methane-air premixed flames. In: Bathe KJ, editor. Computational Fluid and Solid Mechanics. Elsevier, Amsterdam; 2005. p. 650–653.

7. Diamantis DJ, Mastorakos E, Goussis DA. H2/air autoignition: The nature and interaction of the developing explosive modes. Combustion Theory and Modelling. 2015;19(3):382–433.

8. Tingas EA, Im HG, Kyritsis DC, Goussis DA. The use of CO2 as an additive for ignition delay and pollutant control in CH4/air autoignition. Fuel. 2018;211:898–905.

9. Valorani M, Najm HN, Goussis DA. CSP analysis of a transient flame-vortex interaction: time scales and manifolds. Combustion and Flame. 2003;134(1):35–53.

10. Goussis D, Lam S. A study of homogeneous methanol oxidation kinetics using CSP. In: Symposium (International) on Combustion. vol. 24. Elsevier; 1992. p. 113–120.

11. Valorani M, Creta F, Donato F, Najm H, Goussis D. A CSP-based skeletal mechanism generation procedure: auto-ignition and premixed laminar flames in n-heptane/air mixtures. In: ECCOMAS CFD 2006: Proceedings of the European Conference on Computational Fluid Dynamics, Egmond aan Zee, The Netherlands, September 5-8, 2006. Delft University of Technology; European Community on Computational Methods in Applied Sciences (ECCOMAS); 2006.

12. Goussis DA. Quasi steady state and partial equilibrium approximations: their relation and their validity. Combustion Theory and Modelling. 2012;16(5):869–926.
